# Bushen Huoxue recipe restores trophoblast proliferation through the PI3K/AKT pathway in recurrent spontaneous abortion

**DOI:** 10.3389/fmed.2026.1719434

**Published:** 2026-04-21

**Authors:** Jingheng You, Yanqiu Xia, Li Jiang, Hongli Huang, Zhuojun Jiang, Siqing Huang, Li Dong

**Affiliations:** 1 Department of Gynecology, Yueyang Hospital of Integrated Traditional Chinese and Western Medicine, Affiliated Hospital of Shanghai University of Traditional Chinese Medicine, Shanghai, China; 2 Shanghai University of Traditional Chinese Medicine, Shanghai, China

**Keywords:** BSHXR, EVTs, network pharmacology, PI3K/AKT pathway, proliferation, RSA

## Abstract

**Background:**

Dysregulation of extravillous trophoblast (EVT) proliferation and invasion has been implicated in the pathogenesis of recurrent spontaneous abortion (RSA). Bushen Huoxue Recipe (BSHXR), a Traditional Chinese Medicine formula for RSA, has notable clinical efficacy; however, the molecular mechanisms by which it ameliorates recurrent spontaneous abortion remain to be elucidated.

**Objective:**

This study aimed to identify the key active ingredients in BSHXR and observe the mechanism by which BSHXR alleviates RSA.

**Methods:**

BSHXR compounds were identified by UPLC–Q–TOF–MS/MS. Transcriptome sequencing and network pharmacology were performed to identify potential key genes and pathways involved in the therapeutic effects of BSHXR. A miscarriage model was established in mice, and the embryo loss rate, ELISA, and HE staining were used to explore the effect of BSHXR. In combination with bioinformatics analyses, we used immunoblotting and immunofluorescence to elucidate the regulatory mechanisms of BSHXR *in vivo*.

**Results:**

Transcriptome analysis suggests that the pathogenesis of RSA may be associated with cell proliferation and the PI3K/AKT pathway. A total of 155 chemical constituents of BSHXR were identified, along with 267 therapeutic targets, for treating RSA, and the main chemical components of BSHXR stably interact with key genes of the PI3K/AKT pathway. In the BSHXR group, the rate of embryonic loss was significantly reduced (*p* < 0.01), and the levels of serum progesterone (P) and chorionic gonadotropin (CG) increased (*p* < 0.05), with placental tissue morphology tending toward normalcy. Furthermore, the expression of Ki67, PCNA, and proteins related to the PI3K/AKT (*p* < 0.0001) pathway increased in the BSHXR group.

**Conclusion:**

The PI3K/AKT pathway is a potential pathway for RSA intervention. The therapeutic effect of BSHXR is related to the proliferative effect mediated by the PI3K/AKT pathway.

## Graphical abstract

**Figure:**
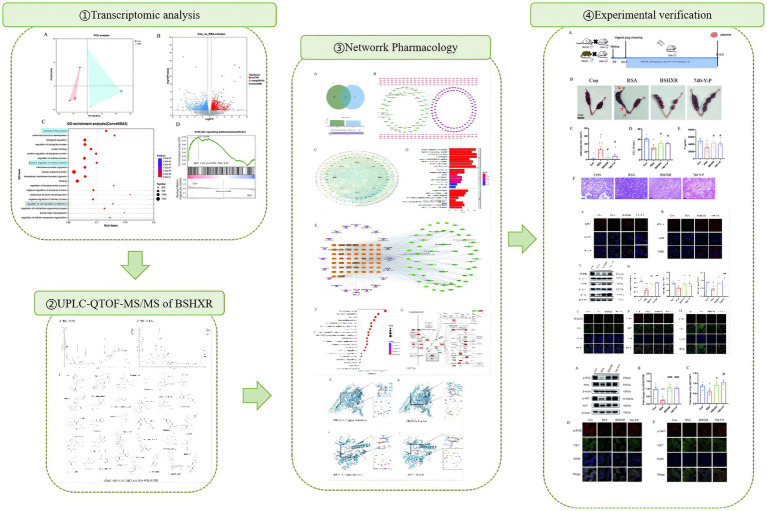

## 1 Introduction

Recurrent spontaneous abortion (RSA) is defined as two or more consecutive spontaneous pregnancy losses before 20 weeks of gestation (1). Abortion is a common pregnancy complication with a global incidence of approximately 10.8% among women of reproductive age, which has a considerable impact on patients’ health and families (2). Despite comprehensive investigations, the underlying cause of RSA remains unexplained in more than 50% of couples (3).

The etiology of RSA is highly heterogeneous, and the established causes include chromosomal abnormalities, uterine anatomical defects, thrombophilia, endocrine disturbances, autoimmune disorders, infectious or inflammatory endometrial conditions, environmental factors, paternal factors, and unexplained RSA (4). Since the underlying pathogenic mechanisms remain incompletely understood, current medical treatments are largely symptomatic (e.g., immunomodulation and hormonal supplementation) and limited by variable efficacy and potential adverse effects (5). Consequently, more effective therapeutic strategies are urgently needed.

Transcriptome sequencing is a widely used high-throughput technology for detecting gene expression in current biomedical research (6). It has facilitated the development of numerous bioinformatics methods and tools for mining transcriptome data, playing a significant role in disease mechanism research, drug development, and personalized medicine of RSA.

According to Traditional Chinese Medicine (TCM) theory, renal deficiency is the root cause of RSA. A deficiency of kidney qi represents dysfunction in the ability of the kidney to consolidate and regulate, and consequently, a deficiency of kidney essence leads to an insufficiency in nourishing the uterine vessels, resulting in repeated pregnancies and repeated abortions.

In recent years, TCM has emerged as a mainstream health care modality and a popular adjunct to conventional therapies for unexplained recurrent spontaneous abortion (URSA) (7, 8). Clinical studies have shown that Chinese herbal formulas focused on kidney-tonifying strategies can significantly increase the ongoing pregnancy rate and live birth rate in RSA patients (9). TCM modulates immune tolerance, cellular proliferation, angiogenesis, and the inflammatory microenvironment through multiple targets (7, 10–12).

Bushen Huoxue Recipe (BSHXR) was developed by the third generation of the State Medical Master Professor Zhu Nansun and has been widely used to treat RSA with marked effects (13, 14). Our previous clinical study revealed that BSHXR significantly increased the 12-week ongoing pregnancy rate in RSA patients, preserved vascular barrier integrity before and after conception, and ameliorated uterine artery blood flow postimplantation (15). However, the chemical basis of this recipe and its potential mechanism remain unknown.

BSHXR is a complex herbal mixture that contains numerous chemical constituents and is presumed to exert therapeutic effects on RSA by simultaneously modulating multiple targets and pathways. Network pharmacology integrates systems biology, pharmacology, and computational science to elucidate the network relationships among herbal constituents of BSHXR, their corresponding targets, metabolic pathways, and RSA processes (16). Molecular docking provides a theoretical framework for predicting receptor–ligand interactions, whereas molecular dynamics (MD) simulation offers a computational platform to explore the *in vivo* motion of small molecules from BSHXR. Both techniques have been extensively used to corroborate network pharmacology findings (17). In recent years, the combination of network pharmacology with molecular docking and MD simulation has become increasingly prevalent in the mechanistic investigation of herbal medicines (18, 19), enabling the identification of putative active constituents of BSHXR and therapeutic targets of RSA for subsequent experimental validation.

**Figure 1:**
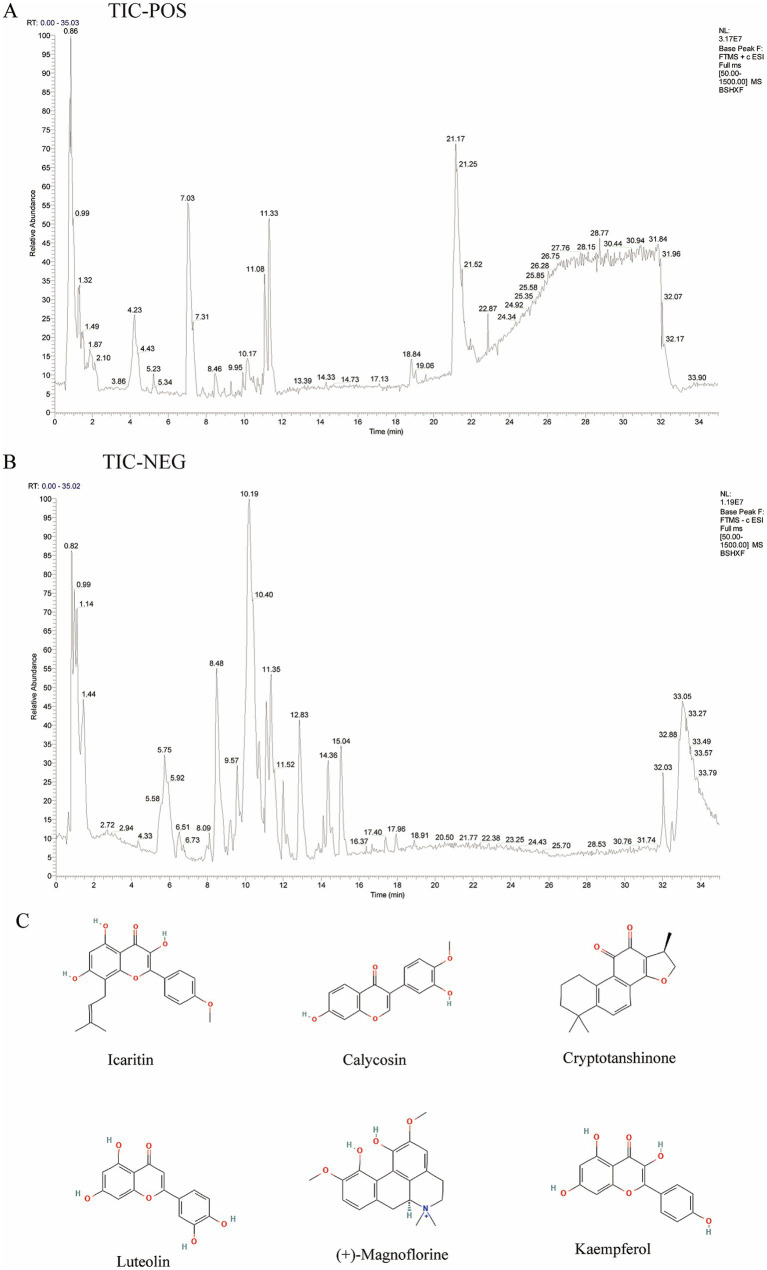
Analysis of the components of BSHXR. **(A,B)** IC of BSHXR in positive-ion and negative-ion modes. **(C)** The main active components of BSHXR.

In this study, we first employed transcriptomic analysis to investigate the potential pathogenesis of RSA in mice. Subsequently, we identified the major components of BSHXR using ultra-high-performance liquid chromatography–quadrupole time-of-flight mass spectrometry/mass spectrometry (UPLC–QTOF–MS/MS). By integrating network pharmacology, molecular docking, and molecular dynamics simulation techniques, we elucidated the potential mechanism by which BSHXR ameliorates RSA. Finally, we experimentally validated the stability and interaction potency of key BSHXR-derived compounds with RSA-relevant protein targets (Figure 1).

## 2 Materials and methods

### 2.1 Reagents and chemicals

740 Y-P was purchased from Selleck, China, Ltd. A mouse chorionic gonadotropin (CG) ELISA kit (ml001975) and a mouse progesterone (P) ELISA kit (ml057778) were purchased from mlbio, China, Ltd. Citrate Antigen Retrieval Solution was supplied by Beyotime, China, Ltd. Antibodies targeting PCNA (13110), Cyclin D1 (55506), p-PI3K (17366), PI3K (4292), and p-AKT (4060) were purchased from Cell Signaling Technology (Danvers, MA, USA). Antibodies against AKT1 (80457-1-RR), β-tubulin (66240-1-Ig), and β-actin (66009-1-Ig) were purchased from Proteintech. Antibodies targeting CDK4 (R023806) and CK7^+^ (M012346) were purchased from Epizyme, China, Ltd. Antibodies against CDK6 (F0372) were supplied by Selleck, China, Ltd. Antibodies against Ki67 (ab16667) were purchased from Abcam (Shanghai, China).

### 2.2 Preparation and composition of BSHXR

BSXHR, in the form of a dry powder, was purchased from Shanghai Wanshicheng National Pharmaceutical Products Co. BSHXR comprises nine traditional Chinese medicinal herbs (Table 1). During the preparation process, the herbs were decocted with water twice and filtered to obtain the extract. The extract was collected, mixed, concentrated, and then purified by the alcohol precipitation method to remove impurities and retain the active ingredients of the drug. Finally, the extract was spray-dried to obtain a dry powder for use.

**Table 1:** Composition of BSHXR.

Chinese name	Original herb name	Latin names	Daily dosage
Ba-Ji-Tian	*Morindae officinalis* How	Morindae officinalis Radix	10 g
Yin-Yang-Huo	*Epimedium brevicornu*	Epimedium brevicornu Maxim	10 g
Dang-Shen	*Codonopsis pilosula* (Franch.) Nannf	Codonopsis Radix	20 g
Dan-Shen	*Salvia miltiorrhiza* Bge	Salviae miltiorrhizae Radix et Rhizoma	15 g
Dang-Gui	*Angelica sinensis* (Oliv.) Diels	Angelicae sinensis Radix	12 g
Huang-Qi	*Astragalus membranaceus* (Fisch) Bge.var.*mongholicus* (Bge.) Hsiao	Astragali Radix	20 g
Shu-Di-Huang	*Rehmannia glutinosa* Libosch.	Rehmanniae Radix Praeparata	15 g
Tu-Si-Zi	*Cuscuta chinensis* Lam.	Cuscutae Semen	12 g
Fu-Pen-Zi	*Rubus chingii* Hu	Rubi Fructus	12 g

### 2.3 Experimental animals

The animal experiments were approved by the Medical Ethics Committee of Shanghai University of Traditional Chinese Medicine (Approval No: PZSHUTCM2507040002) and were carried out in accordance with the Guidelines for the Care and Use of Laboratory Animals. Forty 7-week-old CBA/J female mice (20 g ± 5 g) were purchased from Beijing Huafukang Biotechnology Co. Fifteen 7-week-old DBA/2 J male mice were purchased from Shanghai Slaughter Laboratory Animals Ltd., and five 7-week-old BALB/c male mice were purchased from Beijing Viton Lever Laboratory Animal Technology Co. The experimental animals were kept in the Animal Experiment Center of Shanghai University of Traditional Chinese Medicine [License No. SYXK (Shanghai) 2020–0009] at a room temperature of 22 ± 3 °C, a relative humidity of 50–60%, and light and darkness for 12 h each; the animals were free to eat and drink, and the experiments were started after 1 week of acclimatization.

Forty CBA/J female mice were randomly divided into the Con group, RSA group, BSHXR group, and the 740-Y-P group by the randomized numerical table method. There were 10 mice in each group: 10 CBA/J females were paired with 5 BALB/c males in the Con group; the RSA, BSHXR, and 740-Y-P groups, which consisted of 10 CBA/J mice each, were paired with 15 DBA/2J mice to establish the RSA mouse mode (20). Animal experiments were conducted with each group consisting of six animals. The *n* values for each experiment are indicated in the figure captions.

Mating success was confirmed the next morning at 8 a.m. by observing a vaginal plug or a sperm-positive vaginal smear, and this day was designated as gestational day 0.5 (GD 0.5). From GD 0.5 onward, the BSHXR group mice were based on our team’s prior research (21). The BSHXR group received (182 mg kg^−1^) by gavage once daily, the 740-Y-P group received 740-Y-P (10 mg kg^−1^) by intraperitoneal injection once daily (22), and the Con and RSA groups received equivalent volumes of 0.9% saline by the same routes (Graphical Abstract).

### 2.4 Transcriptome analysis

To identify differentially expressed genes (DEGs) between control and RSA mouse placentae, total RNA was extracted from placental tissues of three mice per group and subjected to high-throughput sequencing on the NovaSeq X Plus platform (Illumina) by Shanghai Majorbio Bio-Pharm Technology Co., Ltd. (Shanghai, China). DEGs were defined as those exhibiting a |log₂-fold change| ≥ 1.5 and *p* ≤ 0.05. Hierarchical clustering of DEGs was performed to delineate gene expression patterns. Gene Ontology (GO) and gene set enrichment analysis (GSEA) were conducted using R (v4.3.2).

### 2.5 Network pharmacology methods

#### 2.5.1 BSXHR component identification and analysis

UPLC–QTOF–MS/MS was used to analyze the main chemical constituents of BSHXR rapidly. The standard solution of each component in BSHXR and the solution of the herbal sample were absorbed. The samples were analyzed under liquid chromatography and TOF/MS mass spectrometry conditions described in the following section to obtain total ion current (TIC) diagrams in both positive and negative ion modes. On the basis of the MassBank database,^1^ the Swiss ADME platform,^2^ and identification and inference through comparisons with relevant literature, unknown compounds in the samples were identified.

Ultrahigh-performance liquid chromatography (UHPLC) was performed on a Waters ACQUITY system equipped with an HSS T3 column (100 mm × 2.1 mm, 1.8 μm) maintained at 40 °C. The mobile phase (Table 2) consisted of acetonitrile (A) and water containing 0.1% formic acid (B). A gradient elution program was used at 0.3 mL min^−1^ with a postcolumn split ratio of 2:1, and the injection volume was 4 μL.

**Table 2:** Mobile phase gradient schedule.

Time (min)	A-water containing 0.1% formic acid (%)	B-acetonitrile (%)
0	98	2
0.5	98	2
2	95	5
3	85	15
13	50	50
25	5	95
30	5	95
30.5	95	5
35	95	5

High-resolution mass spectrometry (HRMS) was conducted on a Q Exactive Orbitrap mass spectrometer (Thermo Fisher Scientific) using heated electrospray ionization (HESI). For positive-ion mode, the parameters used were as follows: ion-source temperature 300 °C, sheath gas 35 arbitrary units (arb), auxiliary gas 15 arb, sweep gas 0 arb, spray voltage 3.8 kV, capillary temperature 350 °C, and S-Lens RF level 30%. For negative-ion mode, the settings were identical except that the sweep gas was 1 arb, the spray voltage was 3.2 kV, and the S-Lens RF level was 60%. Data were acquired in full MS/dd-MS^2^ (TopN = 5) mode over the *m*/*z* range 100–1,500 at a resolving power of 70,000 (FWHM at *m*/*z* 200).

The raw files were processed with Compound Discoverer 3.3 (Thermo Fisher Scientific) using the natural-product template. MS^1^ and MS^2^ spectra were aligned against the OTCML database for compound annotation, applying a mass tolerance of 5 ppm for precursor ions and 10 ppm for fragment ions.

#### 2.5.2 Screening of BSHXR-derived active ingredients and their targets

SMILES strings of all the constituents were retrieved from PubChem and submitted to SwissADME for in silico ADMET profiling. The combined use of these three criteria enables target prediction to focus on molecules that can be absorbed and developed into drugs, while reducing the risk of overinflated network pharmacology results. Only molecules that simultaneously satisfied the following criteria were retained: high gastrointestinal (GI) absorption, Lipinski “Rule of Five” compliance, and fulfilment of the Ghose filter. The combined use of these three criteria enables target prediction to focus on molecules that can be absorbed and developed into drugs, while reducing the risk of overinflated network pharmacology results. The 3D structures of the qualified compounds were then uploaded, together with their SMILES codes, to SwissTargetPrediction (probability >0), SEA (human targets only), and PharmMapper (norm-fit >0.6). The predicted protein identifiers were standardized to official gene symbols via UniProt, and duplicates were removed to yield the final ingredient–target list.

#### 2.5.3 Identification of RSA-related targets

RS-associated genes were mined from GeneCards^3^ with a relevance score >5.182 (median cut-off) and from OMIM^4^ without score filtering. After merging and deduplication, the resulting gene set represented the RSA disease targets.

#### 2.5.4 Intersection of BSHXR and RSA targets

A Venn diagram was generated to visualize the overlap between BSHXR-derived ingredient targets and RSA disease targets. The intersecting genes were loaded into Cytoscape (v3.7.0) for the construction of an ingredient-target network.

#### 2.5.5 PPI network construction and identification of hub genes

The intersecting targets were entered into STRING with the following parameters: combined score ≥0.900 and species limited to “*Homo sapiens*.” The resulting PPI network was visualized using Cytoscape (v3.7.0). Topological centrality analysis was performed; nodes with a degree ≥ 2 × median were defined as hub targets.

#### 2.5.6 Functional enrichment analysis

Hub targets were subjected to Gene Ontology (GO) and Kyoto Encyclopedia of Genes and Genomes (KEGG) enrichment analyses by the clusterProfiler package in R, with “*Homo sapiens*” as the background, and significance was set at *p* < 0.05. The most enriched biological processes and pathways were visualized using the Microbiome Analyst web server.

#### 2.5.7 Construction of the compound–target–pathway network

The top 20 statistically significant KEGG pathways were mapped onto the intersecting targets with Cytoscape (v3.7.0). The resulting network highlighted the key active compounds and pivotal targets through which BSHXR may exert therapeutic effects against RSA.

#### 2.5.8 Molecular docking validation

Representative hub targets (PIK3CA and AKT1) were selected for docking validation. Ligand SDF files were downloaded from PubChem, energy-minimized via ChemDraw 3D (MM2 force field), and exported as mol2 files. Receptor crystal structures were retrieved from the UniProt-linked PDB entries, preprocessed in PyMOL (water removal, hydrogen addition), and converted to pdbqt format using MGLTools (v1.5.6) (charge calculation, merging non-polar hydrogens). Docking simulations were executed with AutoDock Vina (v1.1.2), and the lowest binding energy poses were analyzed visually in PyMOL.

#### 2.5.9 Molecular dynamics simulation

The results of the molecular docking experiments served as the basis for conducting molecular dynamics simulations. The protein was subjected to preprocessing to generate its topological structure. The simulation system was subsequently solvated, energy-minimized, and equilibrated in terms of temperature and pressure. Thereafter, molecular dynamics simulations were initiated.

In this study, an all-atom explicit solvent model was established. The software used was GROMACS (v 2024.05). The system was set at a temperature of 310 K and simulated for a duration of 100 ns. The force field applied to the protein was AMBER99SB-ildn, with the TIP3P model for explicit water molecules. The small molecules were parameterized using the general AMBER force field (GAFF). The atomic charges of the small molecules were calculated using the restrained electrostatic potential (RESP2) method, with the charge calculation performed with ORCA for geometry optimization and MultiF for charge calculation, followed by the generation of force field parameters with Sobtop.

Prior to the MD run, energy minimization was conducted using the steepest descent method for 50,000 steps to eliminate steric clashes. Subsequently, simulations were carried out under the NVT ensemble and NPT (1 bar) ensemble, with each ensemble running for 50,000 steps and 100 ps to equilibrate the system. For pressure regulation, the Berendsen method was employed to maintain the pressure at 1 bar. With respect to temperature control, the V-rescale method was utilized to keep the system temperature at 310 K. Verlet buffer was used to update the neighbor list every 10 steps, with a cut-off distance of 1.2 nm. The entire production process was performed without constraints, with a time step of 2 fs, and trajectory data were saved every 10 ps. The binding free energy and decomposition free energy of the protein–ligand complex were calculated with the gmx_MMPBSA module.

### 2.6 Experimental methods

#### 2.6.1 Embryo loss rate

After 10 days of treatment, the mice were kept fasting and water-free for 12 h, then induced with anesthesia using 5% isoflurane, and subsequently sacrificed by cervical dislocation under deep anesthesia on day 10.5 of gestation. After the placentas were removed, they were stored at −80 °C, and the counts of lost embryos and surviving embryos were documented. The embryo loss rate was calculated according to the following formula: embryo loss rate = number of lost embryos/(number of surviving embryos + number of lost embryos) × 100%. Pictures were taken for intergroup comparisons and then stored at −80 °C.

#### 2.6.2 ELISA

Blood (0.5 mL) was collected from the main abdominal vein of pregnant mice on the 10.5th day of gestation and centrifuged at 3000 rpm and 4 °C for 10 min. Then, 0.1–0.2 mL of the supernatant was collected in a test tube and stored at −80 °C. The levels of P and CG in the serum were measured using ELISA kits, following the manufacturer’s instructions.

#### 2.6.3 Histological examination

Mouse placentas from each group were collected on day 10.5 of gestation, fixed with 4% paraformaldehyde, embedded in paraffin wax, and cut into 5-μm-thick sections, which were then deparaffinized with xylene, washed with anhydrous ethanol, dehydrated with gradient alcohol, and subjected to hematoxylin–eosin (H&E) staining for comparative observation of trophoblast morphological changes under a light microscope.

#### 2.6.4 Immunofluorescence

Five-micron-thick sections of placental tissue were subjected to deparaffinization with xylene, dewaxing, and hydration using gradient alcohol. Sodium citrate antigen repair solution was used for repair in a water bath at 100 °C for 30 min. The sections were blocked with 5% BSA for 15 min and treated with 0.5% tralatone for 1 h. Antibodies directed against Ki67 (1:400), PCNA (1:400), Cyclin D1 (1:100), CDK4 (1:100), CDK6 (1:200), p-PI3K (1:1000), and p-AKT (1:500) were incubated with the tissues overnight at 4 °C. After washing with PBS 3 times, 555 anti-rabbit fluorescent secondary antibodies were added, and the samples were incubated for 1.5 h at room temperature. After incubation for 1.5 h, the samples were washed three times with PBS. Anti-CK7 antibody (1:200) was added, and the samples were incubated at 4 °C overnight, washed with PBS three times, and then incubated with 488 anti-mouse fluorescent secondary antibodies at room temperature for 1.5 h. After washing with PBS three times, the sections were stained with 4′,6-diamidino-2-phenylindole (DAPI) for 10 min and sealed with an anti-fluorescence quencher.

#### 2.6.5 Western blotting

Approximately 10 mg of placental tissue was weighed, the pulp was homogenized, and RIPA lysis solution was added. The mixture was centrifuged at 4 °C and 12,000 rpm for 15 min. The supernatant, which contained total tissue protein, was collected, and its concentration was obtained according to a BCA kit following the manufacturer’s instructions. The proteins were first separated by sodium dodecyl sulfate–polyacrylamide gel electrophoresis (SDS–PAGE) on a 10% gel and then transferred to a polyvinylidene fluoride (PVDF) membrane, which was blocked with 5% skim milk powder, after which it was incubated with antibodies against Cyclin D1 (1:1000), CDK4 (1:1000), CDK6 (1:2000), p-PI3K (1:1000), PI3K (1:1000), p-AKT (1:1000), AKT (1:5000), and β-Tubulin (1:4000) overnight at 4 °C, washed, and then incubated with HRP-labeled secondary antibodies for 1 h at room temperature. An ECL kit was used to detect the proteins, and ImageJ software was used for analysis.

### 2.7 Statistical analysis

The experimental data used in this study were statistically analyzed via GraphPad Prism 8. Data following a normal distribution are presented as the mean ± standard error (*x ± s*). For comparisons among multiple groups, one-way ANOVA was applied. If the variance was homogeneous among groups, the LSD test was used; if the variance was heterogeneous, the Dunnett T3 test was employed. *p* < 0.05 was defined as statistically significant.

## 3 Results

### 3.1 RNA sequencing analysis of potential pathogenic mechanisms in RSA mice

**Figure 2:**
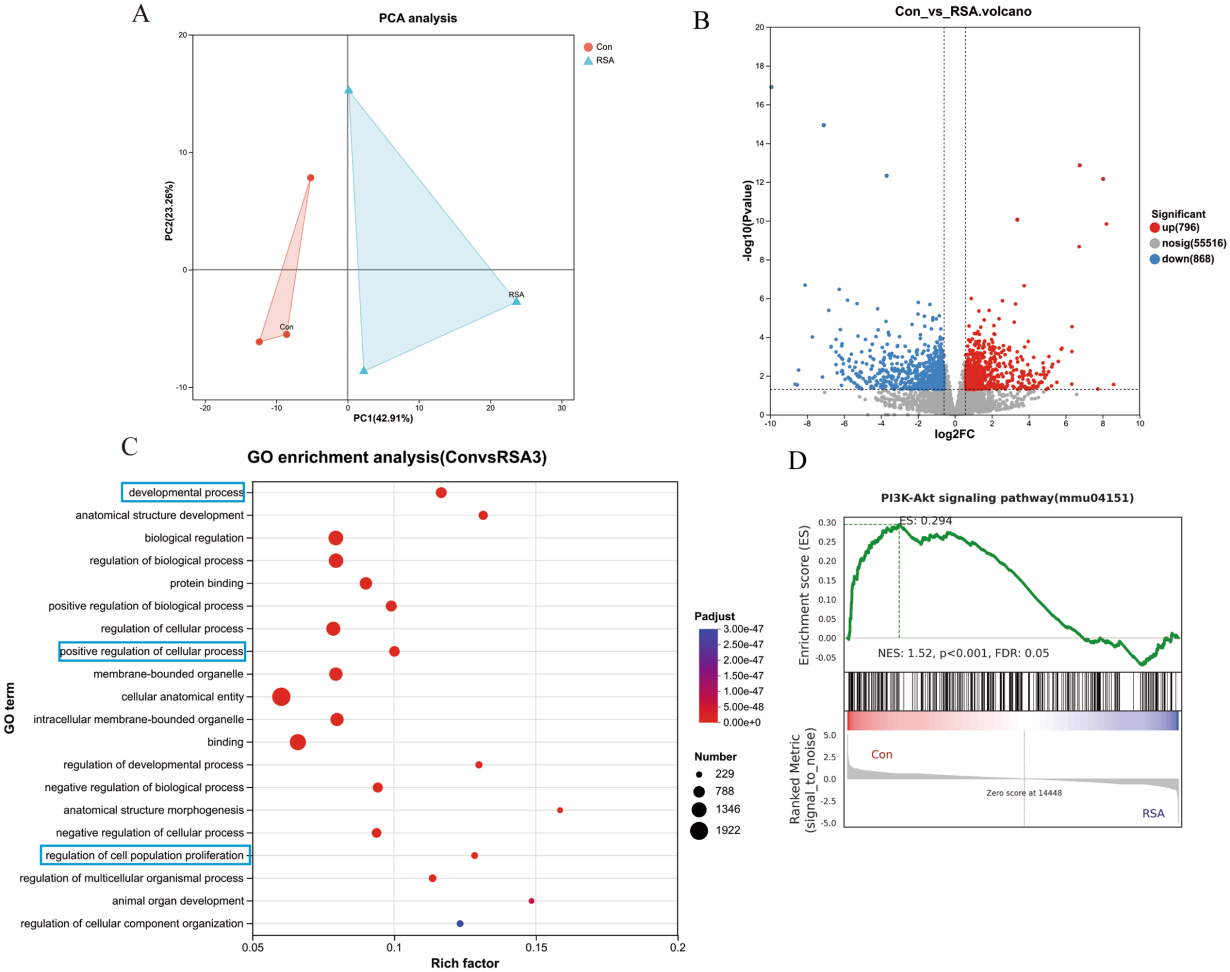
Transcriptional analysis results of CON vs. RSA mice. **(A)** PCA of RNA sequencing data; **(B)** Differential gene volcano maps of CON vs. RSA mice; **(C)** GO enrichment analysis of DEGs between CON and RSA mice; **(D)** GSEA of the PI3K/AKT signaling pathway. *n* = 3.

To investigate the biological mechanisms underlying the occurrence of RSA, the placentas of the mice in the Con and RSA groups were collected for RNA-sequencing analysis. PCA plot revealed that the data of Con and RSA mice were distinctly separated (Figure 2A), indicating significant differences among them. GO enrichment of DEGs in the Con and RSA groups (Supplementary Table S1) revealed significant over-representation of terms related to developmental processes, positive regulation of cellular processes, and regulation of cell population proliferation (Figures 2B,C), and GSEA was also performed on these genes (Figure 2D). The analysis revealed that the PI3K/AKT signaling pathway may be significantly downregulated in RSA.

### 3.2 BSHXR component identification

The chemical constituents of BSHXR were rapidly profiled by UPLC–QTOF–MS/MS, and total-ion-current (TIC) chromatograms were obtained in both positive- and negative-ion modes (Figures 1A,B). A total of 155 characterized compounds were analyzed (Supplementary Table S2). The main chemical constituents identified from BSHXR were confidently or tentatively characterized, with their structures (Figure 1C) and detailed information provided in Table 3, among which two compounds listed in the table were derived from *Epimedium brevicornu* Maxim, and two compounds originated from *Salvia miltiorrhiza* Radix et Rhizoma.

**Table 3:** Main chemical constituents of BSHXR.

No	Name	Formula	*m*/*z*	Traditional Chinese Medicine
1	(+)-Magnoflorine	C20H23NO4	342.17084	*Epimedium brevicornum*
2	Icaritin	C21H20O6	369.13391	*Epimedium brevicornum*
3	Cryptotanshinone	C19H20O3	319.13144	*Salvia miltiorrhiza*
4	Luteolin	C15H10O6	285.03952	*Salvia miltiorrhiza*
5	Kaempferol	C15H10O6	287.05573	*Rubus chingii* Hu
6	Calycosin	C16H12O5	285.07648	*Astragalus membranaceus*

### 3.3 Establishment of network pharmacology for BSHXR components and RSA

**Figure 3:**
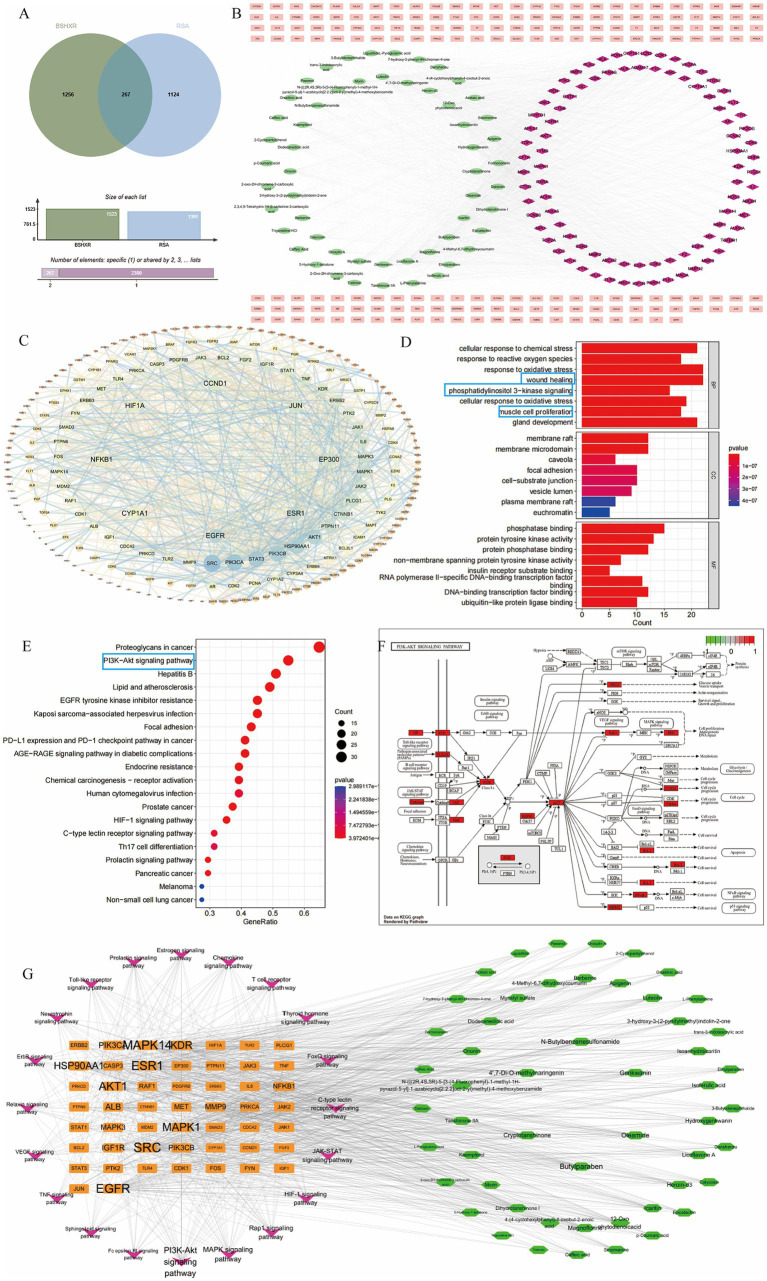
Exploration of potential targets and pathways of BSHXR via network pharmacology: **(A)** Venn diagram of intersecting targets of BSHXR and RSA; **(B)** BSHXR compound–target–RSA network diagram map; **(C)** PPI network diagram with the common targets of BSHXR and RSA; **(D)** GO enrichment analysis; **(E,F)** KEGG enrichment analysis and the target gene–pathway network; **(G)** BSHXR compound–target–pathway network.

The identified active compounds of BSHXR are listed in the Supplementary Table S3. On the basis of these compounds, 1,523 targets were predicted as BSHXR-regulated gene clusters after deduplication. The 1,523 constituent targets of BSHXR were matched with 1,391 RSA-related disease targets, leading to 267 overlapping therapeutic targets, as shown in Figure 3A.

The compound–target–disease network of BSHXR against RSA was constructed using Cytoscape. It consists of 322 nodes (55 active constituents, 267 targets) and 2,364 edges, where each edge denotes an interaction between a compound and its target or a target and RSA (Figure 3B).

**Figure 4:**
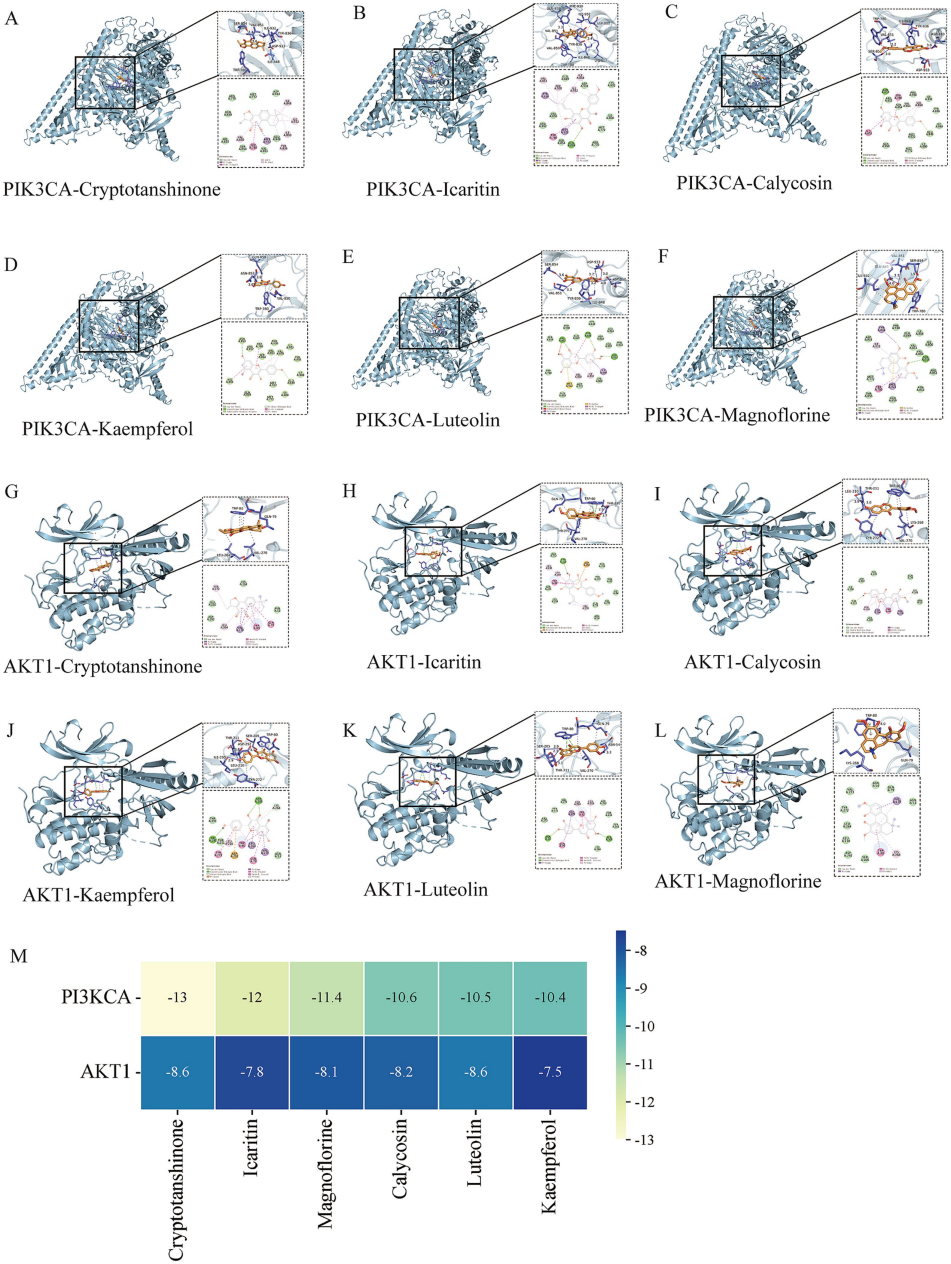
Molecular docking models of the following: **(A)**, Cryptotanshinone with PIK3CA; **(B)**, Icaritin with PIK3CA; **(C)**, Calycosin with PIK3CA; **(D)**, Kaempferol with PIK3CA; **(E)**, Luteolin with PIK3CA; **(F)**, Magnoflorine with PIK3CA; **(G)**, Cryptotanshinone with AKT1; **(H)**, Icaritin with AKT1; **(I)**, Calycosin with AKT1; **(J)**, Kaempferol with AKT1; **(K)**, Luteolin with AKT1; **(L)**, Magnoflorine with AKT1; **(M)**, Heatmap of docking scores between the key chemical constituents of BSHXR and the RSA protein targets.

PPI analysis of the 267 shared targets was performed via STRING (combined score ≥0.900). Topological analysis of the PPI network revealed eight hub genes: EGFR, ESR1, EP300, JUN, CCND1, HIF1A, NFKB1, and CYP1A1 (Figure 3C). Functional enrichment was subsequently conducted. GO analysis revealed 2,479 significantly enriched terms (*p* < 0.05). The top eight entries for each category were visualized as bar plots. Biological processes (BP) were dominated by responses to chemical stress, reactive oxygen species, oxidative stress, wound healing, and muscle cell proliferation. The cellular components (CC) included membrane rafts, caveolae, focal adhesions, cell–substrate junctions, and vesicle lumens. The molecular functions (MF) included phosphatase binding, protein–tyrosine kinase activity, protein–phosphatase binding, non-membrane-spanning protein–tyrosine kinase activity, and insulin–receptor–substrate binding (Figure 3D). KEGG pathway enrichment revealed 171 pathways (*p* < 0.05). The 20 most significant pathways are depicted in Figures 4E,F and include the PI3K/AKT, MAPK, Rap1, HIF-1, and JAK–STAT signaling cascades. The target–pathway network was constructed with Cytoscape (Figure 3G). MAPK1, MAPK14, ESR1, and AKT1 emerged as highly connected nodes, intersecting with the aforementioned pathways. These findings suggest that BSHXR exerts its therapeutic effects on RSA through multitarget modulation of interconnected signaling networks.

### 3.4 Molecular docking analysis of BSHXR and RSA

For further validation of the constituents against RSA-related targets, the top-ranked active constituents of BSHXR were docked into the binding pockets of their corresponding targets (Figures 4A–L). A binding free energy lower than −5.0 kcal mol^−1^ was set as the threshold for reliable hydrophobic interactions (13). All the screened ligand–protein pairs satisfied the criterion, indicating favorable binding (Figure 4M). The docking scores between the constituents of BSHXR and RSA-related targets are listed in Table 4. Notably, icaritin—the representative bioactive constituent of the monarch herb Yin-Yang-Huo—exhibited strong affinity for AKT1 and PIK3CA. These results substantiate the significant binding potential of the key compounds of BSHXR for their predicted target proteins involved in RSA pathogenesis.

**Table 4:** Docking scores between the key chemical constituents of BSHXR and the RSA protein targets.

Receptor name	Ligan name	Scores (kcal/mol)
AKT1	Cryptotanshinone	−13
AKT1	Icaritin	−12
AKT1	Magnoflorine	−11.4
AKT1	Calycosin	−10.6
AKT1	Luteolin	−10.5
AKT1	Kaempferol	−10.4
PI3KCA	Luteolin	−8.6
PI3KCA	Cryptotanshinone	−8.6
PI3KCA	Calycosin	−8.2
PI3KCA	Magnoflorine	−8.1
PI3KCA	Icaritin	−7.8
PI3KCA	Kaempferol	−7.5

### 3.5 Molecular dynamics simulation analysis of BSHXR and RSA

**Figure 5:**
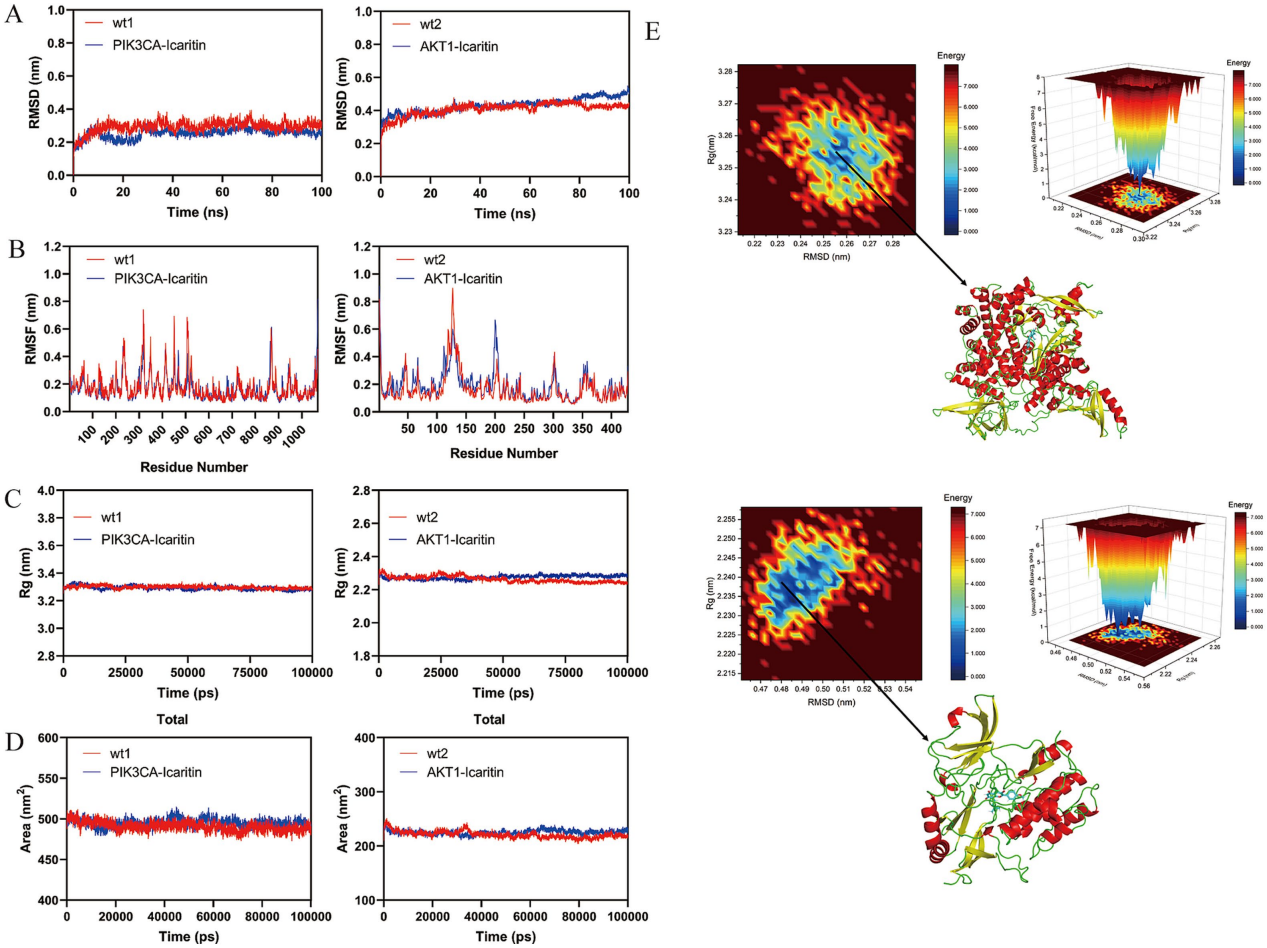
Molecular dynamics simulation of BSHXR and RSA. **(A)** RMSD plot of PIK3CA–icaritin and AKT1–icaritin; **(B)** RMSF plot of PIK3CA–icaritin and AKT1–icaritin; **(C)** Rg plot of PIK3CA–icaritin and AKT1–icaritin; **(D)** SASA plot of PIK3CA–icaritin and AKT1–icaritin; **(E)** Gibbs free energy landscape of PIK3CA–icaritin and AKT1–icaritin.

According to the results of molecular docking, icaritin, along with PIK3CA and AKT1, the key proteins in the PI3K/AKT pathway, with binding energies ≤ −7 kcal/mol, were selected for further molecular dynamics simulation. The results revealed that the binding conformations of PIK3CA–icaritin and AKT1–icaritin remained stable throughout the 100-ns simulation period, with the former stabilizing after 20 ms and the latter stabilizing after 10 ns (Figure 5A), suggesting relatively stable binding interactions. The RMSF measures the fluctuation of each amino acid residue in the protein over the simulation time. In this study, the binding pocket of PI3KCA fluctuated less throughout the simulation, whereas the binding pocket of AKT1 fluctuated more than that of PI3KCA, yet the protein–ligand binding pockets of both remained stable (Figure 5B). Rg reflects the compactness of the molecular structure. In this study, the Rg of PIK3CA–icaritin gradually decreased, indicating that during the simulation, the binding of the ligand to the protein promoted compactness of the overall conformation, whereas the Rg of AKT1–icaritin was slightly greater than that of the wild type (wt2), indicating that the binding of the ligand may have altered the local conformation of the protein binding pocket, leading to increased dynamics in the loop regions and a slight increase in Rg (Figure 5C). The SASA results revealed that during the molecular dynamics simulation, the solvent-accessible surface area (SASA) of the wild-type protein, PIK3CA–icaritin, and AKT1–icaritin remained relatively stable, but the SASA of the complexes was slightly greater than that of the wild-type proteins, which may be related to conformational changes induced by ligand binding (Figure 5D). The free energy landscape plot drawn from the RMSD and Rg trajectories of the last 10 ns of a 100-ns simulation shows that the blue area in the 2D plot indicates the lowest free energy point for the binding of PIK3CA–icaritin and AKT1–icaritin(Figure 5E), indicating that the PIK3CA–icaritin and AKT1–icaritin complexes at this site are the most stable conformations with the lowest free energy throughout the entire simulation process. On the basis of the results of the molecular dynamics simulation, the stability and dynamic interaction between icaritin, a key constituent of BSHXR, and PIK3CA and AKT1, key proteins of the PI3K/AKT signaling pathway, were verified.

### 3.6 BSHXR improves the pregnancy outcome of RSA mice

**Figure 6:**
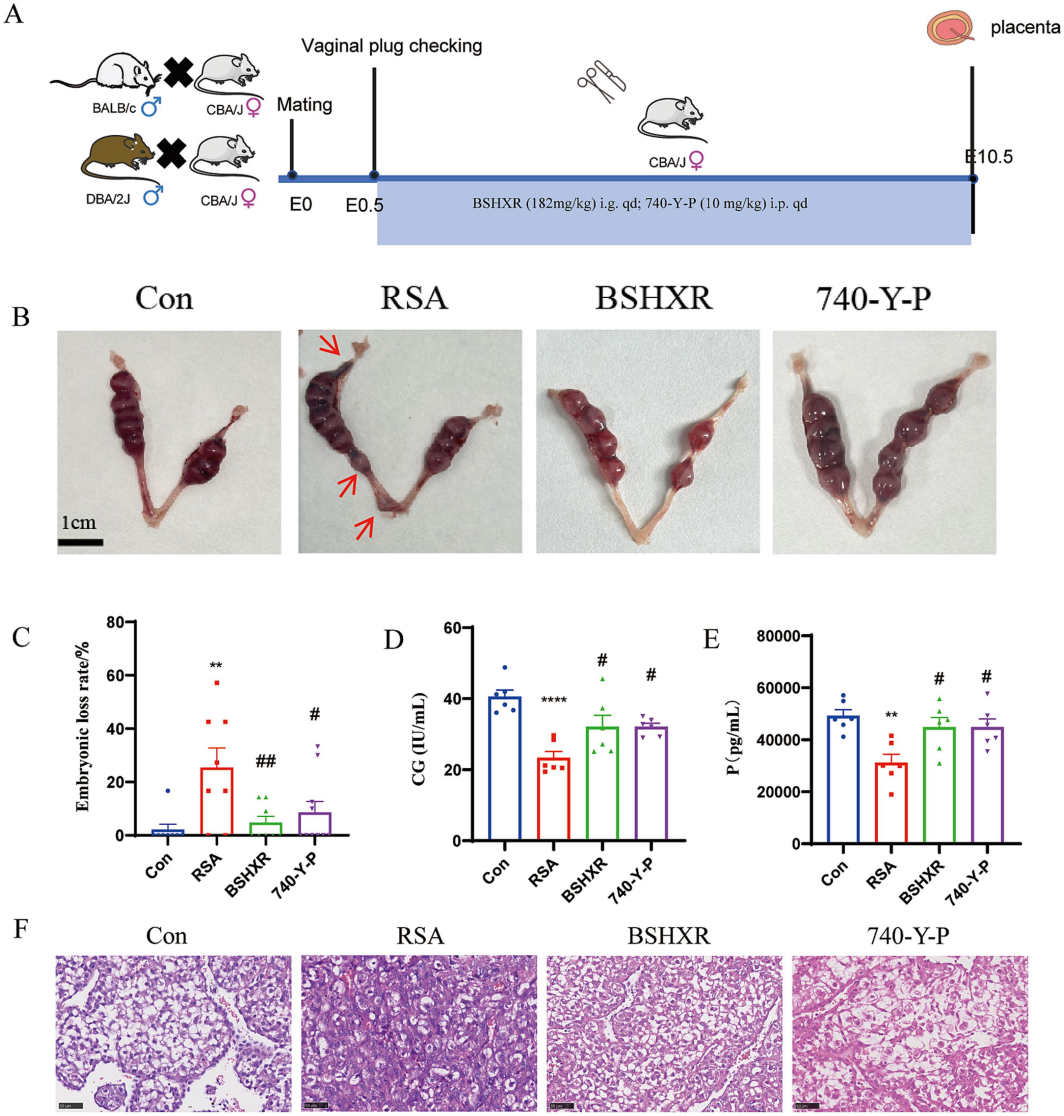
BSHXR has significant therapeutic effects on RSA. **(A)** Schematic diagram of the mode of drug administration in the mouse model. **(B)** Uterus of the mice in each group. Red arrows indicate resorbed embryos. **(C)** Embryo loss rate of the mice in each group, *n* = 6. **(D,E)** Serum P and CG levels of the mice in each group. Note that the red arrows indicate resorbed embryos, *n* = 6. Con: control group; RSA: recurrent spontaneous abortion; BSHXR: Bushen Huoxue recipe; 740-Y-P: agonist of the PI3K pathway. **(F)** Morphological changes in the uterine metaphase tissue of the mice in each group (400×). Scale bar = 50 μm. ^*^p < 0.05, ^**^p < 0.01, ^***^p < 0.001, ^****^p < 0.0001 vs. the Con group; ^#^*p* < 0.05, ^##^*p* < 0.01, ^###^*p* < 0.001, ^####^*p* < 0.0001 vs. the RSA group. The data represent the mean ± SEM.

To validate the predictions from transcriptomics and network pharmacology, *in vivo* experiments were conducted using RSA mouse models. In the control group, the uterine embryos appeared robust and bead-like, with a uniform size and a plump morphology. The embryos exhibited pale red coloration, and no apparent hemorrhage spot was observed within the uterine cavity. In the RSA group, the embryos displayed significant size heterogeneity, with some showing a noticeable reduction in size or a complete absence. Compared with those of the control group, the coloration of the embryos was darker, and evident hemorrhagic foci were present within the uterine cavity. The abnormal morphology of embryos in RSA mice was reversed by BSHXR and 740-Y-P (Figure 6B).

Compared with that in the control group, the embryo loss rate was significantly greater in the RSA group (*p* < 0.01), whereas the rate of embryo loss was significantly lower in the BSHXR (*p* < 0.01) and 740-Y-P groups (*p* < 0.05) than in the RSA group (Figure 6C). The serum P (*p* < 0.0001) and CG (*p* < 0.01) levels were significantly lower in the RSA group than in the control group but significantly increased after treatment with BSHXR (*p* < 0.05) or 740-Y-P (*p* < 0.05) (Figures 6D,E).

Compared with the clear and intact decidual tissue of the control group, the RSA group presented a decrease in decidual cells, a disordered arrangement, some condensed or missing nuclei, uneven cytoplasmic staining, damaged vascular endothelial cells, incomplete vessel walls, and significant interstitial oedema. In contrast, the decidual cell morphology and structure were significantly normalized in the BSHXR and 740-Y-P groups (Figure 6F). Our findings indicate that BSHXR can improve pregnancy outcomes in RSA mice.

### 3.7 Validation of the effects of BSHXR on proliferation

**Figure 7:**
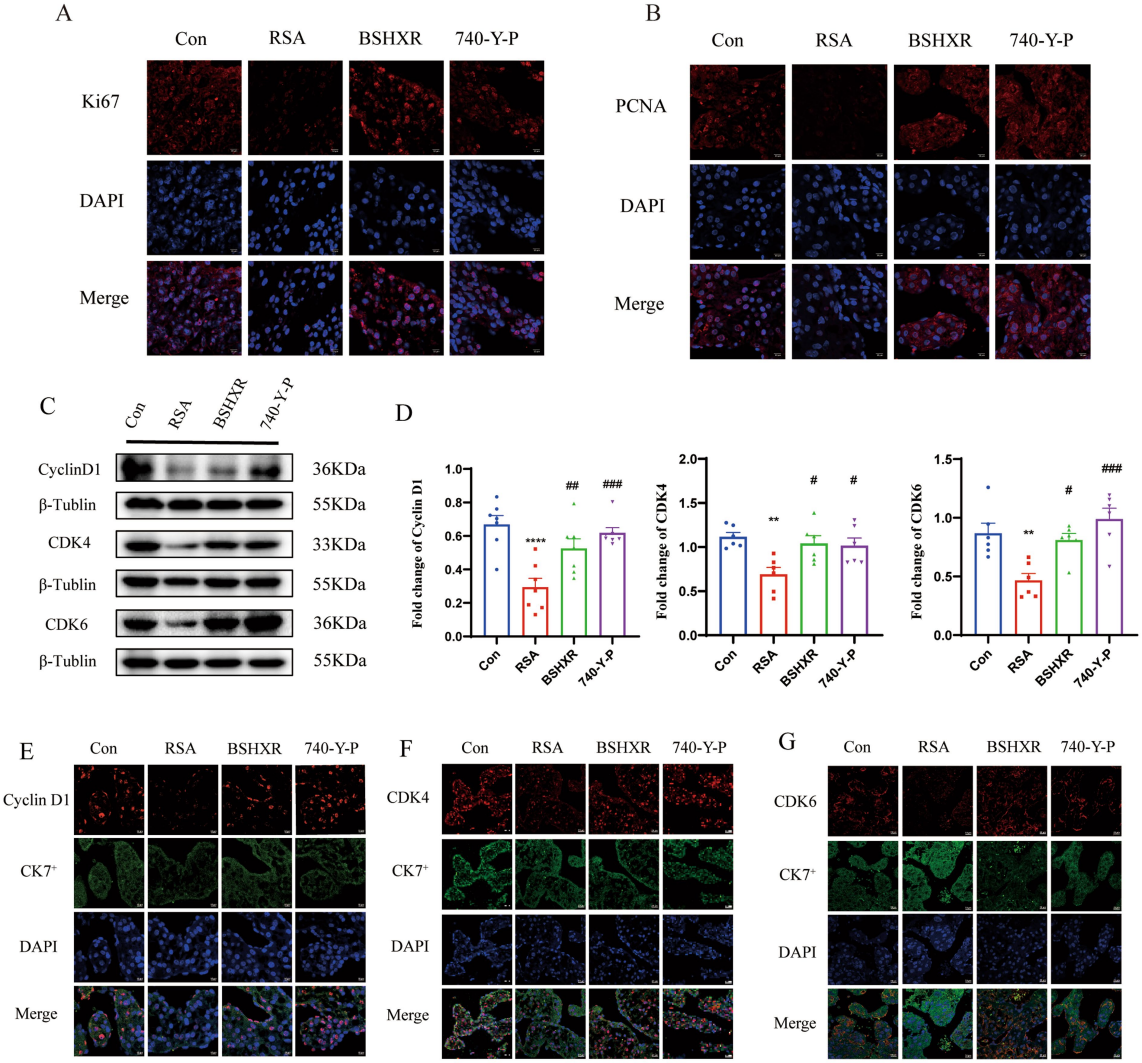
Proliferation of placental tissues from mice. **(A,B)** Immunofluorescence of Ki67 and PCNA in placental tissues. **(C,D)** The protein expression of CyclinD1, CDK4, and CDK6 in placental tissues, detected by western blotting, *n* = 6; **(E–G)** Immunofluorescence of the indicated proteins. Scale bar = 20 μm. ^*^p < 0.05, ^**^p < 0.01, ^***^p < 0.001, ^****^p < 0.0001 vs. the Con group; ^#^*p* < 0.05, ^##^*p* < 0.01, ^###^*p* < 0.001, ^####^*p* < 0.0001 vs. the RSA group. The data represent the mean ± SEM.

The network pharmacology and transcriptomic GO enrichment analysis of this study suggest that BSHXR may treat RSA by restoring cellular proliferation capacity. The nuclear antigens Ki67 and PCNA—both established regulators of the cell cycle (23)—were markedly altered, underscoring their central roles in proliferative events. Immunofluorescence staining of placental sections revealed that Ki-67 and PCNA protein levels were significantly elevated in both the BSHX group and the 740-Y-P group (Figures 7A,B), indicating marked restoration of proliferative activity. According to the PPI network (Figure 3C), CCND1 (Cyclin D1) emerged as a hub gene whose primary function is the regulation of the cell cycle. Cyclin D1 forms specific complexes with CDK4 and CDK6, thereby governing the G1/S transition—a checkpoint that is critically modulated by BSHXR treatment. Compared with those in the control group, the protein expression levels of CyclinD1 (*p* < 0.0001), CDK4 (*p* < 0.01), and CDK6 (*p* < 0.01) were significantly lower in the RSA group but were significantly greater in response to BSHXR (*p* < 0.05) or 740-Y-P (*p* < 0.05) treatment (Figures 7C,D). Immunofluorescence of CylinD1, CDK4, and CDK6 also verified these results (Figures 7E–G).

### 3.8 BSHXR improves pregnancy outcomes by regulating the PI3K/AKT pathway

**Figure 8:**
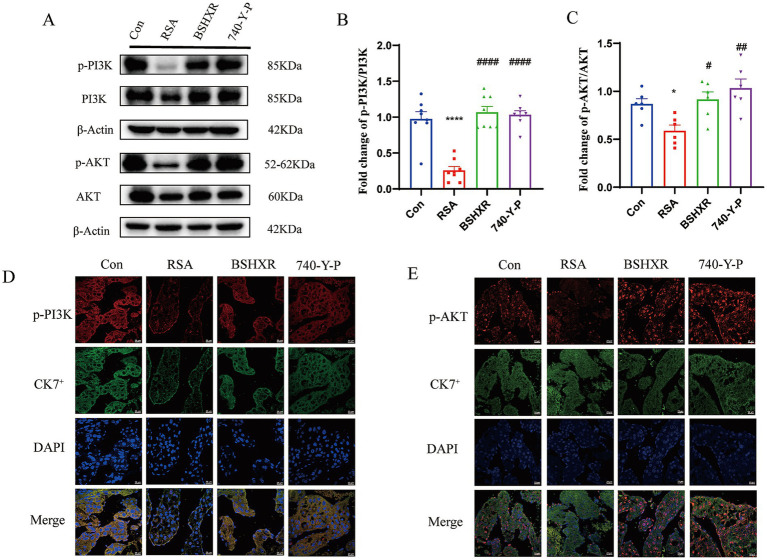
Expression of p-PI3K and p-AKT in placental tissues. **(A–C)** The expression of p-PI3K and p-AKT in the placental tissues was detected by western blotting, *n* = 6; **(D,E)** IF of p-PI3K and p-AKT in placental tissues. Scale bar = 20 μm. ^*^p < 0.05, ^**^p < 0.01, ^***^p < 0.001, ^****^p < 0.0001 vs. the on group; ^#^*p* < 0.05, ^##^*p* < 0.01, ^###^*p* < 0.001, ^####^*p* < 0.0001 vs. the cRSA group. The data represent the mean ± SEM.

To further investigate the regulatory mechanism of BSHXR, we evaluated the main target proteins of the PI3K/AKT pathway. Compared with those in the control group, the protein expression levels of p-PI3K (*p* < 0.0001) and p-AKT (*p* < 0.05) were significantly lower in the RSA group but increased in BSHXR (*p* < 0.05) and 740-Y-P (*p* < 0.01) groups (Figures 8A–C). IF of p-PI3K and p-AKT also verified these results (Figures 8D,E).

## 4 Discussion

RSA is a prevalent disorder among women of reproductive age, and its incidence continues to rise annually, imposing substantial emotional and economic burdens on affected families. TCM has shown promising clinical efficacy in RSA management, offering individualized therapy with minimal toxicity (24).

Among the multifactorial etiologies of RSA, impaired biological function of EVTs has emerged as a critical pathogenic node (25). EVTs, located at the outermost layer of the mature embryo, mediate embryo–maternal recognition and are indispensable for embryo implantation, placental development, and pregnancy maintenance. The highly invasive and proliferative nature of EVTs is essential for early gestation (26). Reduced EVT proliferation and invasion are recognized as key histopathological features of RSA. Thus, restoring EVT proliferative and invasive capacity has become a central therapeutic focus.

Our previous study demonstrated that BSHXR improves RSA outcomes in mice by normalizing the maternal–fetal vascular microenvironment and reducing thrombosis (27). Icaritin, the main active ingredient in Yin-Yang-Huo, can enhance pregnancy outcomes in RSA mice by regulating the Treg/Th17 imbalance through the TGF-β/SMAD signaling pathway (12). According to previous studies (28), *Cuscuta* functions by regulating sex hormones and also has effects such as enhancing immunity and combating fatigue. However, the precise molecular mechanisms by which BSHXR restores normal EVT function remain to be elucidated.

Accumulating evidence indicates that the PI3K/AKT signaling pathway, which orchestrates cell growth, proliferation, metabolism, and survival, critically modulates the proliferative and invasive capacities of EVTs and is intimately linked to the pathogenesis of RSA (10). FKBP5, an inhibitor of the PI3K/AKT pathway, has been reported to impair trophoblast function (29).The heterodimeric enzyme PI3K is composed of two subunits: the regulatory subunit p85 and the catalytic subunit p110, which is encoded by the PIK3CA gene (30). AKT is a crucial downstream effector molecule of the PI3K pathway. Among the three isoforms of AKT, AKT1 is expressed in most tissues and is the core member most abnormally activated during cancer proliferation (31). The activation of PI3K can lead to the phosphorylation of AKT, thereby regulating cell growth, proliferation, and differentiation (32). According to our PPI network analysis of BSHXR-responsive genes, CCND1 and EGFR were identified as pivotal targets. EGFR, a receptor tyrosine kinase, governs trophoblast differentiation, proliferation, migration, and invasion (33). Its upregulation potently activates the PI3K/AKT cascade in trophoblasts (34). Cyclin D1, a downstream cell cycle regulator of PI3K/AKT, together with cyclin E2, CDK2, CDK4, and pRb, effectively halts the cell cycle and inhibits the proliferation of breast cancer cells (35). Downregulation of these proteins halts the cell cycle and inhibits proliferation, whereas decidual tissues from women with unexplained RSA exhibit markedly reduced expression of cyclin D, CDK4, and CDK6 (36).

**Figure 9:**
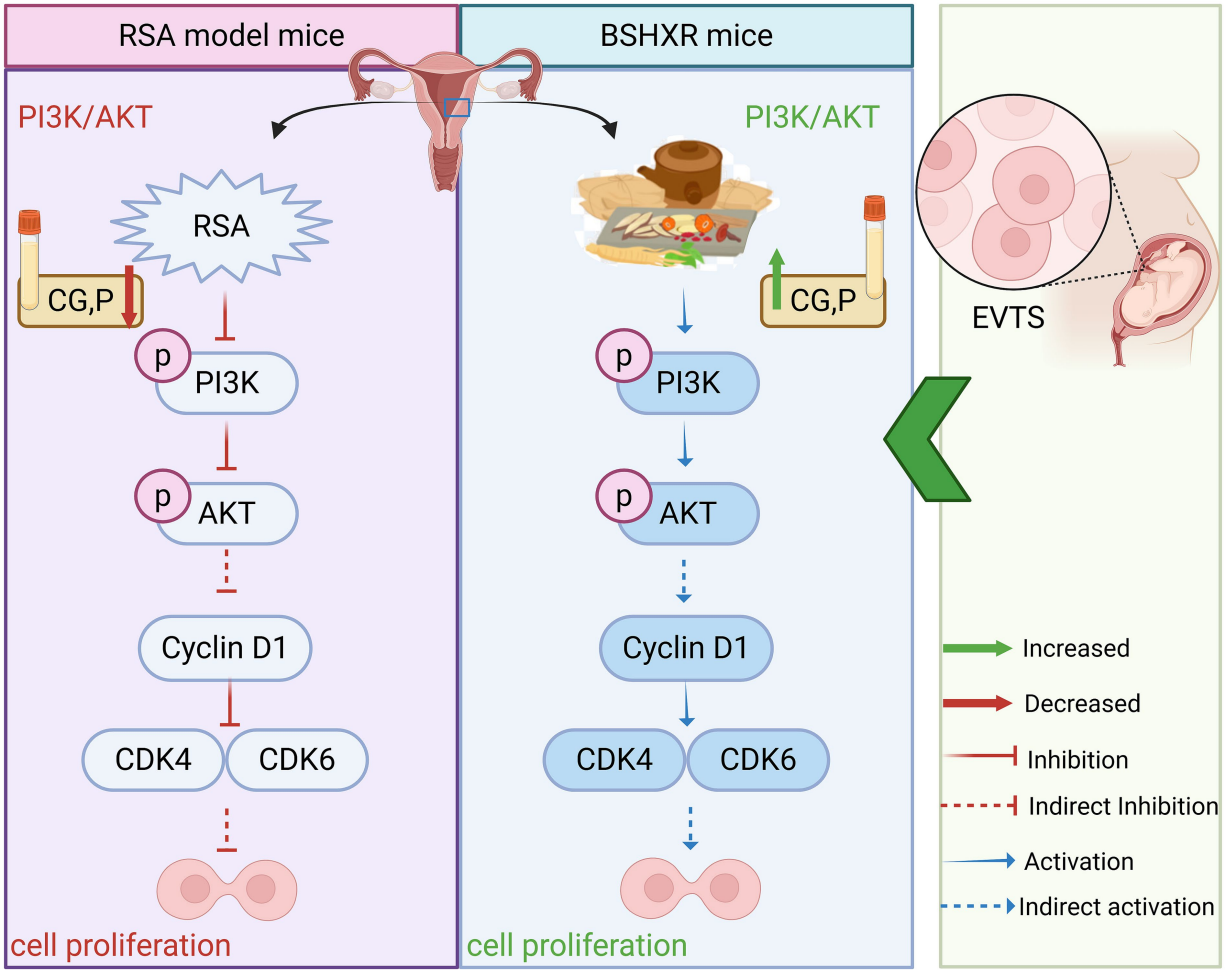
Molecular mechanisms of BSHXR in RSA.

This study integrates transcriptomics and network pharmacology findings, both of which point to cell proliferation as the core phenotype. Transcriptomic GSEA revealed significant downregulation of the PI3K/AKT gene set in the RSA model, and KEGG analysis in network pharmacology showed the highest enrichment level for the PI3K/AKT pathway. Based on this evidence, we employed molecular docking and MD to validate whether the main component of BSHXR improves RSA via the PI3K/AKT pathway. Ultimately, the network pharmacology-predicted target and transcriptomically measured differentially expressed genes jointly identified the “PI3K/AKT-cell proliferation” axis as the core mechanism by which BSHXR ameliorates RSA, providing experimental rationale for subsequent functional validation. Finally, we further validated the predicted mechanism through *in vivo* experiments (Figure 9).

This study integrated UPLC–QTOF–MS/MS, RNA-seq, network pharmacology, and MD simulation. Through multidimensional evidence corroborating each other, it conclusively identified the PI3K/AKT–cell proliferation axis as a verifiable target pathway for BSHXR intervention in RSA, laying a mechanistic foundation for designing small molecules or biological agents. But molecular docking was performed only for the six most abundant constituents. The remaining compounds were not examined individually. In addition, transcriptome analysis failed to yield more effective data on target genes due to the limited amount of placental tissue available.

## 5 Conclusion

In this study, BSHXR was demonstrated to enhance the proliferative capacity of compromised trophoblasts in RSA mice and upregulate the PI3K/AKT signaling pathway in mouse placental tissue. Our findings provide a robust theoretical basis for the broader clinical application of BSHXR in the management of RSA.

## Data Availability

The raw sequencing data generated in this study have been deposited in the NCBI Sequence Read Archive (SRA) and can be accessed using accession number PRJNA1436952. Data access link: https://www.ncbi.nlm.nih.gov/sra/PRJNA1436952.

## Footnotes

1
https://massbank.eu/MassBank/

2
https://www.swissadme.ch/

3
https://www.genecards.org/

4
https://www.omim.org/

## Glossary

BSHXR: Bushen Huoxue Recipe
EVT: extravillous TROPHOBLAST
RSA: recurrent spontaneous abortion
URSA: unexplained recurrent spontaneous abortion
UPLC–Q–TOF–MS/MS: Ultra Performance Liquid Chromatography–Quadrupole Time-of-Flight Mass Spectrometry/Mass Spectrometry
PCNA: Proliferating Cell Nuclear Antigen
PI3K: Phosphatidylinositol 3-kinases
AKT: Protein Kinase B
p-PI3K: Phosphorylated Phosphatidylinositol 3-kinases
PIK3CA: Phosphatidylinositol-4,5-Bisphosphate 3-Kinase Catalytic Subunit Alpha
AKT1: Serine/Threonine-Protein Kinase 1
p-AKT: Phosphorylated protein kinase B
740-Y-P: 740 Y-PDGFR
CDK4: Cyclin-Dependent Kinase 4
CDK6: Cyclin-Dependent Kinase 6
CK7^+^: Cytokeratin 7 Positive
GD: gestational day
Con: Control
RMSD: Root Mean Square Deviation
RMSF: Root Mean Square Fluctuation
Rg: Radius of Gyration
SASA: solvent-accessible surface area
