# Correction: Bushen Huoxue recipe restores trophoblast proliferation through the PI3K/AKT pathway in recurrent spontaneous abortion

**DOI:** 10.3389/fmed.2026.1866883

**Published:** 2026-05-22

**Authors:** Jingheng You, Yanqiu Xia, Li Jiang, Hongli Huang, Zhuojun Jiang, Siqing Huang, Li Dong

**Affiliations:** 1 Department of Gynecology, Yueyang Hospital of Integrated Traditional Chinese and Western Medicine, Affiliated Hospital of Shanghai University of Traditional Chinese Medicine, Shanghai, China; 2 Shanghai University of Traditional Chinese Medicine, Shanghai, China

**Keywords:** BSHXR, EVTs, network pharmacology, PI3K/AKT pathway, proliferation, RSA

There was a mistake in Graphic Abstract and Figure 7 as published. Due to a lack of careful review, two sets of images for CDK6 immunofluorescence were accidentally duplicated. Since the article includes a Graphical Abstract, which needs to be corrected as well. The corrected Figure 7 and Graphical Abstract appears below.

**Figure 7:**
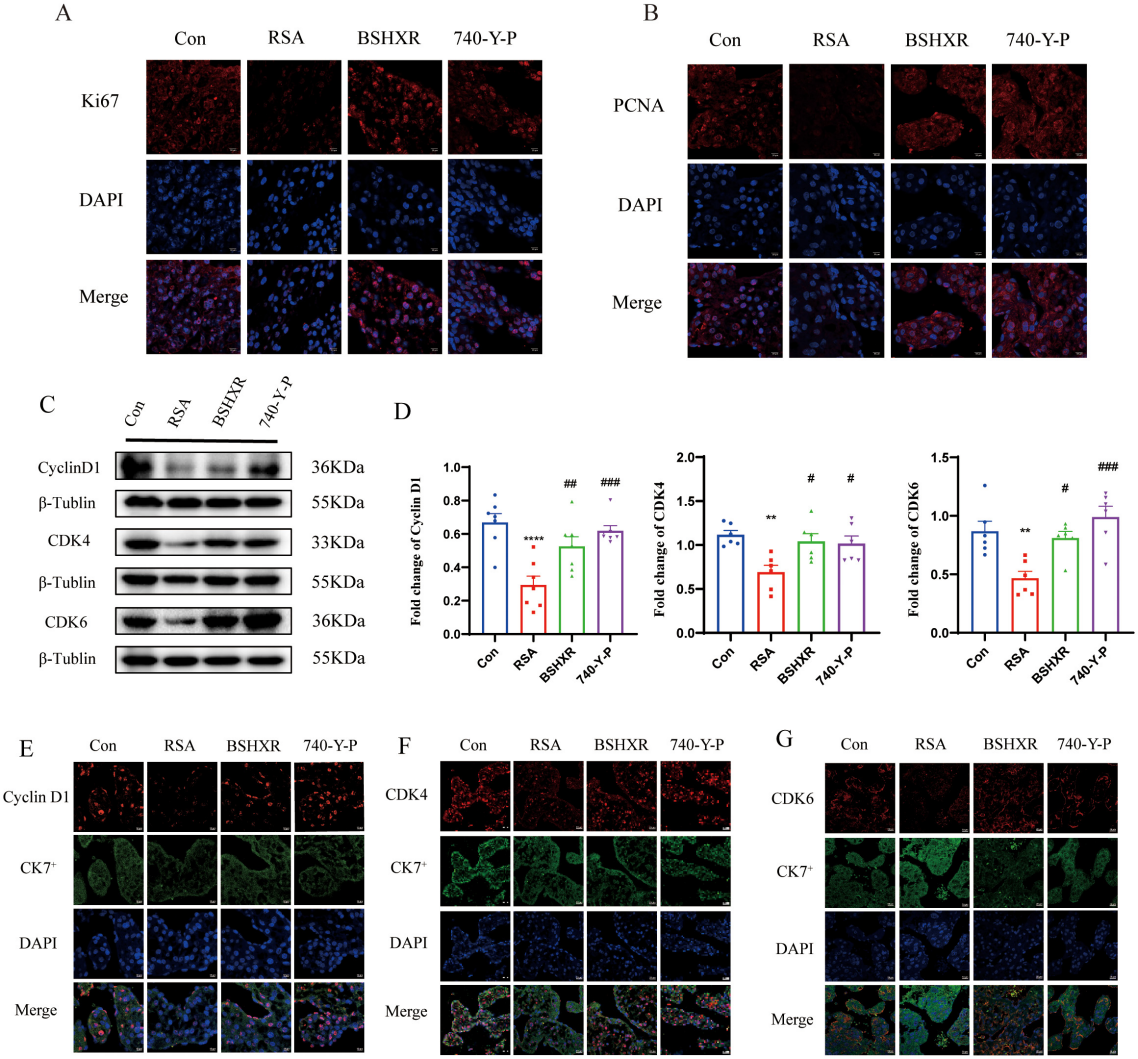
Proliferation of placental tissues from mice. (A, B) Immunofluorescence of Ki67 and PCNA in placental tissues. (C, D) The protein expression of CyclinD1, CDK4, and CDK6 in placental tissues, detected by western blotting, n = 6; (E–G) Immunofluorescence of the indicated proteins. Scale bar = 20μm. *p < 0.05, **p < 0.01, ***p < 0.001, ****p < 0.0001 vs. the Con group; #p < 0.05, ##p < 0.01, ###p < 0.001, ####p < 0.0001 vs. the RSA group. The data represent the mean ± SEM.

**Graphical abstract:**
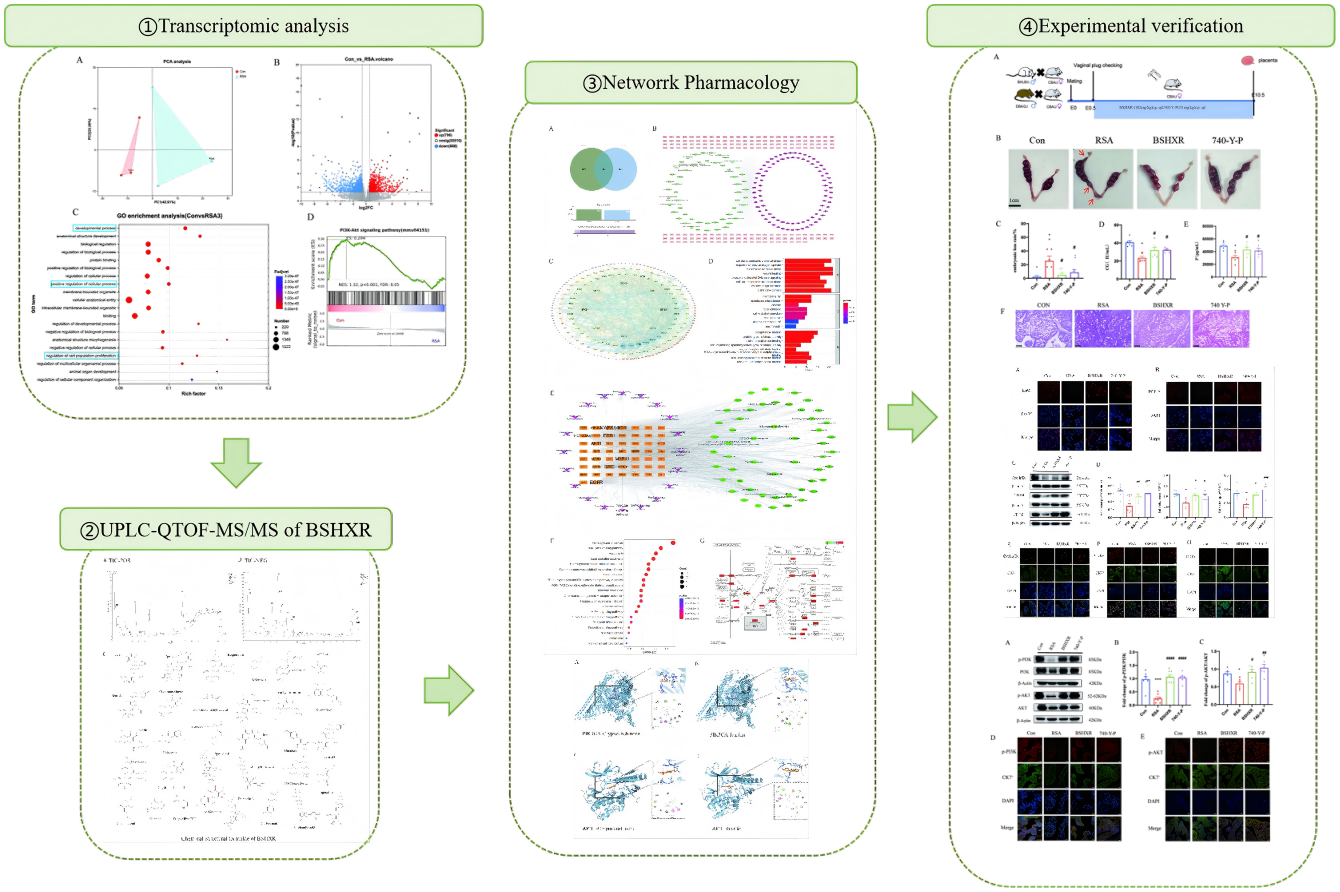


The original version of this article has been updated.

