# Supplementary material for: Bushen Huoxue recipe restores trophoblast proliferation through the PI3K/AKT pathway in recurrent spontaneous abortion
Source: Front Med (Lausanne). 2026 Apr 21;13:1719434. doi: 10.3389/fmed.2026.1719434 (PMC13139346; doi:10.3389/fmed.2026.1719434)
Supplement: Supplementary file 1 [file Table_1.docx]

| No | Mode | Name | Formula | m/z | Area |
| --- | --- | --- | --- | --- | --- |
| 1 | ESI+2 | (+)-Magnoflorine | C20 H23 N O4 | 342.2 | 27611191 |
| 2 | ESI- | (1S,3R,4R,5R)-1,3,4-trihydroxy-5-{[(2E)-3-(4-hydroxy-3-methoxyphenyl)prop-2-enoyl]oxy}cyclohexane-1-carboxylic acid | C17 H20 O9 | 367.1 | 2154717 |
| 3 | ESI- | (3β,5ξ,9ξ)-3,23-Dihydroxy-1-oxoolean-12-en-28-oic acid | C30 H46 O5 | 485.3 | 1308345 |
| 4 | ESI- | 12-Oxo phytodienoic acid | C18 H28 O3 | 291.2 | 2862794 |
| 5 | ESI+ | 2,3,4,9-Tetrahydro-1H-β-carboline-3-carboxylic acid | C12 H12 N2 O2 | 217.1 | 7825822 |
| 6 | ESI- | 2-Cyclopentylphenol | C11 H14 O | 161.1 | 3652522 |
| 7 | ESI+ | 2-Hydroxyquinoline | C9 H7 N O | 146.1 | 3668172 |
| 8 | ESI+ | 2-Mercaptoethanol | C2 H6 O S | 79.0 | 3522390 |
| 9 | ESI- | 2-Methylbenzoic acid | C8 H8 O2 | 135.0 | 11570046 |
| 10 | ESI+ | 2-oxo-2H-chromene-3-carboxylic acid | C10 H6 O4 | 191.0 | 4054646 |
| 11 | ESI+ | 2-oxopiperidine-3-carbohydrazide | C6 H11 N3 O2 | 158.1 | 13215542 |
| 12 | ESI+ | 2-Pyrrolidinecarboxylic acid | C5 H9 N O2 | 116.1 | 3.06E+08 |
| 13 | ESI- | 3-(tert-Butyl)-N-[4-(2,3-dihydroimidazo[2,1-b][1,3]thiazol-6-yl)phenyl]-1-methyl-1H-pyrazole-5-carboxamide | C20 H23 N5 O S | 380.2 | 1375503 |
| 14 | ESI- | 3,4-Dihydroxyphenylethanol | C8 H10 O3 | 153.1 | 1784250 |
| 15 | ESI+ | 3-Butylidenephthalide | C12 H12 O2 | 189.1 | 6435939 |
| 16 | ESI+ | 3-hydroxy-3-(2-pyridylmethyl)indolin-2-one | C14 H12 N2 O2 | 241.1 | 17895205 |
| 17 | ESI- | 3-Hydroxy-3-methylglutaric acid | C6 H10 O5 | 207.1 | 20126310 |
| 18 | ESI+ | 4-(2,3-dihydro-1H-indol-1-yl)-1-phenyl-1H-pyrazolo[3,4-d]pyrimidine | C19 H15 N5 | 314.1 | 2859200 |
| 19 | ESI- | 4-(4-cyclohexylphenyl)-4-oxobut-2-enoic acid | C16 H18 O3 | 257.1 | 1964830 |
| 20 | ESI+ | 4',7-Di-O-methylnaringenin | C17 H16 O5 | 301.1 | 4286284 |
| 21 | ESI+ | 4-Benzyl-2-({4-[5-(trifluoromethyl)-2-pyridinyl]piperazino}methyl) | C22 H27 F3 N4 O | 421.2 | 5048240 |
| 22 | ESI+ | 4-Ethynylaniline | C8 H7 N | 118.1 | 12139517 |
| 23 | ESI- | 4-Hydroxybenzaldehyde | C7 H6 O2 | 121.0 | 7716059 |
| 24 | ESI+ | 4-Indolecarbaldehyde | C9 H7 N O | 146.1 | 12185768 |
| 25 | ESI+ | 4-Methyl-6,7-dihydroxycoumarin | C10 H8 O4 | 193.1 | 12518174 |
| 26 | ESI- | 4-Methylumbelliferyl glucuronide | C16 H16 O9 | 351.1 | 3194658 |
| 27 | ESI- | 4-Oxoproline | C5 H7 N O3 | 128.0 | 45857051 |
| 28 | ESI+ | 5-Hydroxy-1-tetralone | C10 H10 O2 | 342.2 | 3.28E+08 |
| 29 | ESI+ | 5-Hydroxymethylfurfural | C6 H6 O3 | 127.0 | 79820248 |
| 30 | ESI+ | 6-Methoxyquinoline | C10 H9 N O | 160.1 | 9949005 |
| 31 | ESI+ | 6-Methylquinoline | C10 H9 N | 144.1 | 10548701 |
| 32 | ESI+ | 7-chloro-4-[(5-propyl-2-pyrimidinyl)oxy]quinazoline | C15 H13 Cl N4 O | 301.1 | 3748736 |
| 33 | ESI- | 7-hydroxy-3-phenyl-4H-chromen-4-one | C15 H10 O3 | 237.1 | 1427351 |
| 34 | ESI- | 7-Hydroxycoumarin | C9 H6 O3 | 161.0 | 3248343 |
| 35 | ESI+ | Acetophenone | C8 H8 O | 121.1 | 10528399 |
| 36 | ESI+ | Adenine | C5 H5 N5 | 136.1 | 12320900 |
| 37 | ESI+ | Adenosine 3'5'-cyclic monophosphate | C10 H12 N5 O6 P | 330.1 | 5173539 |
| 38 | ESI+ | Adenosine | C10 H13 N5 O4 | 268.1 | 92241017 |
| 39 | ESI- | Apigenin | C15 H10 O5 | 269.0 | 1771698 |
| 40 | ESI- | Arjungenin | C30 H48 O6 | 503.3 | 1724061 |
| 41 | ESI- | Asiatic acid | C30 H48 O5 | 487.3 | 5979582 |
| 42 | ESI+ | Asparagine | C4 H8 N2 O3 | 133.1 | 10488910 |
| 43 | ESI- | Asperulosidic acid | C18 H24 O12 | 431.1 | 2523310 |
| 44 | ESI- | Azelaic acid | C9 H16 O4 | 187.1 | 8560585 |
| 45 | ESI- | Baohuoside I | C27 H30 O10 | 513.2 | 61254401 |
| 46 | ESI+ | Berberine | C20 H17 N O4 | 336.1 | 6754032 |
| 47 | ESI+ | Bis(2-ethylhexyl)adipate | C22 H42 O4 | 371.3 | 7107923 |
| 48 | ESI- | Butylparaben | C11 H14 O3 | 193.1 | 3773030 |
| 49 | ESI- | Caffeic acid | C9 H8 O4 | 179.0 | 17223502 |
| 50 | ESI+ | Calycosin | C16 H12 O5 | 285.1 | 18045651 |
| 51 | ESI+ | Calycosin-7-O-β-D-glucoside | C22 H22 O10 | 447.1 | 13553679 |
| 52 | ESI+ | Chlorogenic acid | C16 H18 O9 | 377.1 | 5252806 |
| 53 | ESI+ | Choline | C5 H13 N O | 104.1 | 1.5E+08 |
| 54 | ESI+ | Crotonic acid | C4 H6 O2 | 104.1 | 21624676 |
| 55 | ESI- | Cryptochlorogenic acid | C16 H18 O9 | 353.1 | 37502047 |
| 56 | ESI+ | Cryptotanshinone | C19 H20 O3 | 319.1 | 81140666 |
| 57 | ESI- | Cynaroside | C21 H20 O11 | 447.1 | 4394095 |
| 58 | ESI- | D-(-)-Quinic acid | C7 H12 O6 | 191.1 | 2869329 |
| 59 | ESI+ | D-(+)-Pyroglutamic Acid | C5 H7 N O3 | 130.0 | 1.46E+08 |
| 60 | ESI- | Daidzein | C15 H10 O4 | 253.0 | 4651577 |
| 61 | ESI- | Danshensu | C9 H10 O5 | 197.0 | 38846133 |
| 62 | ESI+ | Dihydrotanshinone I | C18 H14 O3 | 301.1 | 28635011 |
| 63 | ESI+ | Dioctyl phthalate | C24 H38 O4 | 391.3 | 18035591 |
| 64 | ESI+ | DL-Arginine | C6 H14 N4 O2 | 175.1 | 2.21E+08 |
| 65 | ESI+ | DL-Glutamine | C5 H10 N2 O3 | 147.1 | 10880900 |
| 66 | ESI+ | DL-Stachydrine | C7 H13 N O2 | 144.1 | 6355421 |
| 67 | ESI- | Dodecanedioic acid | C12 H22 O4 | 229.1 | 1090699 |
| 68 | ESI- | Emodin-3-methyl ether/Physcion | C16 H12 O5 | 283.1 | 5407519 |
| 69 | ESI- | Epicatechin | C15 H14 O6 | 289.1 | 4615815 |
| 70 | ESI+ | Epimedin A1 | C39 H50 O20 | 839.3 | 20116631 |
| 71 | ESI+ | Epmedin B | C38 H48 O19 | 809.3 | 28531582 |
| 72 | ESI+ | Epmedin C | C39 H50 O19 | 823.3 | 1.25E+08 |
| 73 | ESI- | Esculetin | C9 H6 O4 | 177.0 | 4669879 |
| 74 | ESI+ | Ethylparaben | C9 H10 O3 | 167.1 | 1285603 |
| 75 | ESI+ | Formononetin | C16 H12 O4 | 269.1 | 10557932 |
| 76 | ESI- | Formononetin | C16 H12 O4 | 267.1 | 10467207 |
| 77 | ESI- | Forsythoside E | C20 H30 O12 | 461.2 | 2011953 |
| 78 | ESI- | Galactonic acid | C6 H12 O7 | 195.1 | 18048015 |
| 79 | ESI- | Geniposidic acid | C16 H22 O10 | 373.1 | 3431344 |
| 80 | ESI- | Genkwanin | C16 H12 O5 | 283.1 | 1582112 |
| 81 | ESI- | Glucuronic acid-3,6-lactone | C6 H8 O6 | 175.0 | 3441982 |
| 82 | ESI+ | Guanine | C5 H5 N5 O | 152.1 | 26178050 |
| 83 | ESI+ | Guanosine | C10 H13 N5 O5 | 284.1 | 9020631 |
| 84 | ESI+ | Heroin-d3 | C21 H20 [2]H3 N O5 | 373.2 | 13260436 |
| 85 | ESI+ | Hydroxygenkwanin | C16 H12 O6 | 301.1 | 814713.2 |
| 86 | ESI+ | Hyperoside | C21 H20 O12 | 487.1 | 30451613 |
| 87 | ESI+ | Icariin | C33 H40 O15 | 677.2 | 1.8E+08 |
| 88 | ESI+ | Icarisid I | C27 H30 O11 | 531.2 | 87283231 |
| 89 | ESI+ | Icaritin | C21 H20 O6 | 369.1 | 40995285 |
| 90 | ESI- | Icaritin | C21 H20 O6 | 367.1 | 1360489 |
| 91 | ESI- | Isoacteoside | C29 H36 O15 | 623.2 | 7596844 |
| 92 | ESI+ | Isoanhydroicaritin | C21 H20 O6 | 369.1 | 18221918 |
| 93 | ESI+ | Isoferulic acid | C10 H10 O4 | 195.1 | 10089396 |
| 94 | ESI- | Isoguanosine | C10 H13 N5 O5 | 282.1 | 4190743 |
| 95 | ESI- | Isomucronulatol 7-O-glucoside | C23 H28 O10 | 463.2 | 1442420 |
| 96 | ESI- | Isoquercitrin | C21 H20 O12 | 463.1 | 1.21E+08 |
| 97 | ESI- | Kaempferitrin | C27 H30 O14 | 577.2 | 2495318 |
| 98 | ESI+ | Kaempferol | C15 H10 O6 | 287.1 | 10972934 |
| 99 | ESI- | Kaempferol-7-O-β-D-glucopyranoside | C21 H20 O11 | 447.1 | 38640350 |
| 100 | ESI+ | Kojic acid | C6 H6 O4 | 143.0 | 23303136 |
| 101 | ESI- | L-(-)-Malic acid | C4 H6 O5 | 133.0 | 1.41E+08 |
| 102 | ESI+ | L-(+)-Arginine | C6 H14 N4 O2 | 175.1 | 76045623 |
| 103 | ESI- | L-(+)-Lactic acid | C3 H6 O3 | 179.1 | 21251787 |
| 104 | ESI+ | L-Aspartic acid | C4 H7 N O4 | 134.0 | 1963247 |
| 105 | ESI+ | L-Canavanine | C5 H12 N4 O3 | 177.1 | 4990848 |
| 106 | ESI+ | L-Glutamic acid | C5 H9 N O4 | 148.1 | 4389472 |
| 107 | ESI+ | Licoflavone A | C20 H18 O4 | 323.1 | 13159821 |
| 108 | ESI+ | Ligustilide | C12 H14 O2 | 191.1 | 12014733 |
| 109 | ESI+ | Lithospermic acid | C27 H22 O12 | 539.1 | 28155051 |
| 110 | ESI+ | L-Leucine | C6 H13 N O2 | 132.1 | 1.01E+08 |
| 111 | ESI- | Loganic acid | C16 H24 O10 | 375.1 | 2560659 |
| 112 | ESI- | Lonicerin | C27 H30 O15 | 593.1 | 6498544 |
| 113 | ESI+ | L-Phenylalanine | C9 H11 N O2 | 166.1 | 48879268 |
| 114 | ESI+ | L-Pyroglutamic acid | C5 H7 N O3 | 130.0 | 11297178 |
| 115 | ESI- | L-Tryptophan | C11 H12 N2 O2 | 203.1 | 7728481 |
| 116 | ESI- | Luteolin | C15 H10 O6 | 285.0 | 6678034 |
| 117 | ESI+ | L-Valine | C5 H11 N O2 | 118.1 | 15919961 |
| 118 | ESI- | Maleic acid | C4 H4 O4 | 115.0 | 6165734 |
| 119 | ESI- | Malonic acid | C3 H4 O4 | 103.0 | 5404285 |
| 120 | ESI- | Mannitol | C6 H14 O6 | 217.0 | 3734999 |
| 121 | ESI+ | Morin | C15 H10 O7 | 303.1 | 55325935 |
| 122 | ESI+ | morpholine-4-carboximidamide hydrobromide | C5 H11 N3 O | 130.1 | 37263930 |
| 123 | ESI- | Myristyl sulfate | C14 H30 O4 S | 293.2 | 6778585 |
| 124 | ESI- | N-({(2R,4S,5R)-5-[3-(4-Fluorophenyl)-1-methyl-1H-pyrazol-5-yl]-  1-azabicyclo[2.2.2]oct-2-yl}methyl)-4-methoxybenzamide | C26 H29 F N4 O2 | 483.2 | 2721399 |
| 125 | ESI+ | N-Butylbenzenesulfonamide | C10 H15 N O2 S | 214.1 | 74854323 |
| 126 | ESI+ | Neochlorogenic acid | C16 H18 O9 | 377.1 | 19662570 |
| 127 | ESI+ | Nicotinic acid | C6 H5 N O2 | 124.0 | 10458082 |
| 128 | ESI+ | Nipecotic acid | C6 H11 N O2 | 130.1 | 13456894 |
| 129 | ESI+ | Octyl hydrogen phthalate | C16 H22 O4 | 279.2 | 11937190 |
| 130 | ESI+ | Oleamide | C18 H35 N O | 282.3 | 2619776 |
| 131 | ESI+ | Ononin | C22 H22 O9 | 431.1 | 12856000 |
| 132 | ESI- | Ornithine | C5 H12 N2 O2 | 131.1 | 3202926 |
| 133 | ESI- | Oroxylin A | C16 H12 O5 | 283.1 | 2468400 |
| 134 | ESI- | Orsellinic acid | C8 H8 O4 | 167.0 | 27057572 |
| 135 | ESI+ | Paeonol | C9 H10 O3 | 167.1 | 1523439 |
| 136 | ESI- | p-Coumaric acid | C9 H8 O3 | 163.0 | 5816504 |
| 137 | ESI- | Phenol | C6 H6 O | 93.0 | 6128709 |
| 138 | ESI- | Phenylacetaldehyde | C8 H8 O | 119.1 | 5347792 |
| 139 | ESI+ | Polydatin | C20 H22 O8 | 391.1 | 1868803 |
| 140 | ESI- | Protocatechuic acid | C7 H6 O4 | 153.0 | 4198021 |
| 141 | ESI- | Quinic acid | C7 H12 O6 | 191.1 | 6007970 |
| 142 | ESI- | Rosmarinic acid | C18 H16 O8 | 359.1 | 5740899 |
| 143 | ESI- | Salicylic acid | C7 H6 O3 | 137.0 | 1.36E+08 |
| 144 | ESI- | Salvianolic acid C | C26 H20 O10 | 537.1 | 16646736 |
| 145 | ESI+ | Sinomenine | C19 H23 N O4 | 330.2 | 7784318 |
| 146 | ESI- | Stachyose | C24 H42 O21 | 665.2 | 5643561 |
| 147 | ESI- | Sucrose | C12 H22 O11 | 377.1 | 51042811 |
| 148 | ESI+ | Tanshinone IIA | C19 H18 O3 | 317.1 | 23950737 |
| 149 | ESI+ | trans-3-Indoleacrylic acid | C11 H9 N O2 | 188.1 | 53520985 |
| 150 | ESI- | Tretinoin | C20 H28 O2 | 299.2 | 1239899 |
| 151 | ESI+ | Trigonelline HCl | C7 H7 N O2 | 138.1 | 24855470 |
| 152 | ESI- | Ursolic acid | C30 H48 O3 | 455.4 | 923708.1 |
| 153 | ESI- | Verbascoside | C29 H36 O15 | 623.2 | 1882590 |
| 154 | ESI- | Vicenin II | C27 H30 O15 | 593.1 | 3019145 |
| 155 | ESI+ | α-Linolenic acid | C18 H30 O2 | 279.2 | 6688305 |
