# Supplementary material for: Bushen Huoxue recipe restores trophoblast proliferation through the PI3K/AKT pathway in recurrent spontaneous abortion
Source: Front Med (Lausanne). 2026 Apr 21;13:1719434. doi: 10.3389/fmed.2026.1719434 (PMC13139346; doi:10.3389/fmed.2026.1719434)
Supplement: Supplementary file 2 [file Table_2.docx]

| Degree | Eccentricity | Name |
| --- | --- | --- |
| 90 | 3 | Genkwanin |
| 77 | 3 | Licoflavone A |
| 76 | 3 | Magnoflorine |
| 74 | 3 | Butylparaben |
| 72 | 3 | Icaritin |
| 70 | 4 | Oleamide |
| 67 | 4 | Hydroxygenkwanin |
| 67 | 3 | Cryptotanshinone |
| 66 | 4 | Isoanhydroicaritin |
| 66 | 3 | Heroin-d3 |
| 66 | 3 | 12-Oxo-phytodienoicacid |
| 65 | 4 | Luteolin |
| 65 | 3 | 4',7-Di-O-methylnaringenin |
| 63 | 4 | Morin |
| 59 | 4 | Apigenin |
| 58 | 3 | N-Butylbenzenesulfonamide |
| 57 | 4 | Kaempferol |
| 56 | 4 | Dodecanedioic acid |
| 54 | 4 | Ononin |
| 53 | 3 | 3-hydroxy-3-(2-pyridylmethyl)indolin-2-one |
| 50 | 3 | Berberine |
| 49 | 3 | Calycosin |
| 48 | 4 | Oroxylin A |
| 45 | 4 | Myristyl sulfate |
| 43 | 4 | Tanshinone IIA |
| 41 | 5 | L-Phenylalanine |
| 40 | 3 | Ethylparaben |
| 40 | 3 | 4-Methyl-6,7-dihydroxycoumarin |
| 39 | 5 | Isoferulic acid |
| 39 | 4 | Epicatechin |
| 39 | 3 | Dihydrotanshinone I |
| 35 | 3 | Daidzein |
| 34 | 5 | Sinomenine |
| 34 | 4 | N-({(2R,4S,5R)-5-[3-(4-Fluorophenyl)-1-methyl-1H-pyrazol-5-yl]-1-azabicyclo[2.2.2]oct-2-yl}methyl)-4-methoxybenzamide |
| 34 | 3 | Formononetin |
| 34 | 4 | Danshensu |
| 34 | 4 | Azelaic acid |
| 34 | 4 | 4-(4-cyclohexylphenyl)-4-oxobut-2-enoic acid |
| 31 | 5 | L-Pyroglutamic acid |
| 31 | 3 | 7-hydroxy-3-phenyl-4H-chromen-4-one |
| 30 | 5 | Ligustilide |
| 29 | 5 | 3-Butylidenephthalide |
| 28 | 5 | trans-3-Indoleacrylic acid |
| 25 | 4 | Paeonol |
| 25 | 4 | Orsellinic acid |
| 23 | 5 | Caffeic acid |
| 22 | 5 | 2-Cyclopentylphenol |
| 20 | 5 | p-Coumaric acid |
| 20 | 5 | 2-oxo-2H-chromene-3-carboxylic acid |
| 11 | 5 | Trigonelline HCl |
| 11 | 5 | Tretinoin |
| 8 | 5 | 5-Hydroxy-1-tetralone |
| 7 | 5 | 2-Oxo-2H-chromene-3-carboxylic acid |
| 1 | 1 | 2,3,4,9-Tetrahydro-1H-carboline-3-carboxylic acid |
