# Supplementary material for: Bushen Huoxue recipe restores trophoblast proliferation through the PI3K/AKT pathway in recurrent spontaneous abortion
Source: Front Med (Lausanne). 2026 Apr 21;13:1719434. doi: 10.3389/fmed.2026.1719434 (PMC13139346; doi:10.3389/fmed.2026.1719434)
Supplement: Supplementary file 3 [file Table_3.docx]

**WB original gels**


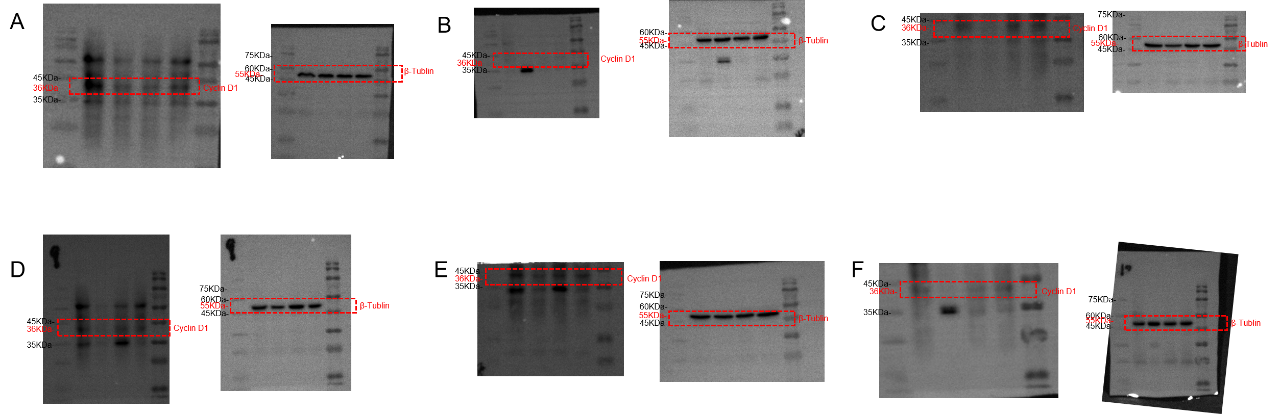
The raw data of CyclinD 1 & β-Tublin in Figure 7D. A. The original image of CyclinD 1 and β-Tublin in Figure 7D; B-F: Raw data of CDK4 and β-Tublin in Figure 7D.


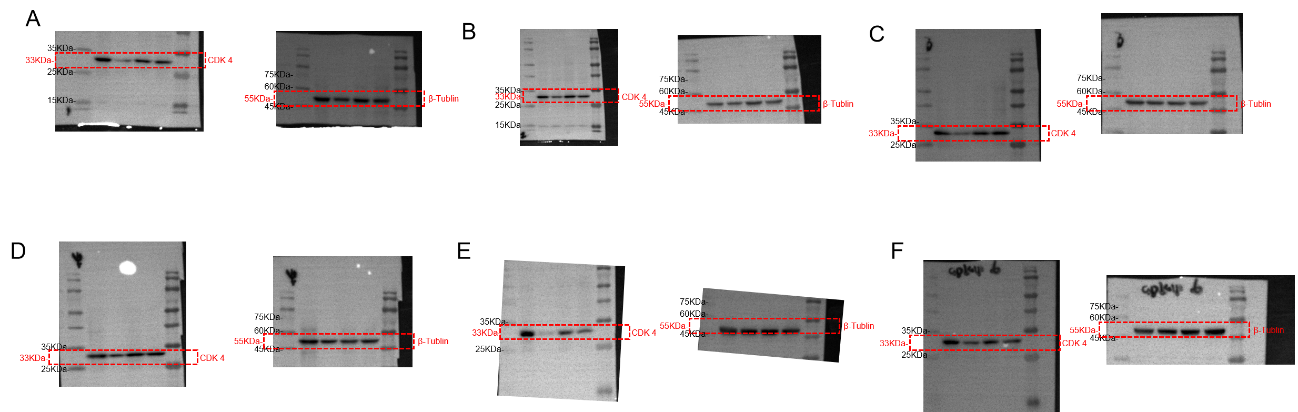


The raw data of CDK4 & β-Tublin in Figure 7D. A. The original image of CDK 4 and β-Tublin in Figure 7D; B-F: Raw data of CDK4 and β-Tublin in Figure 7D.


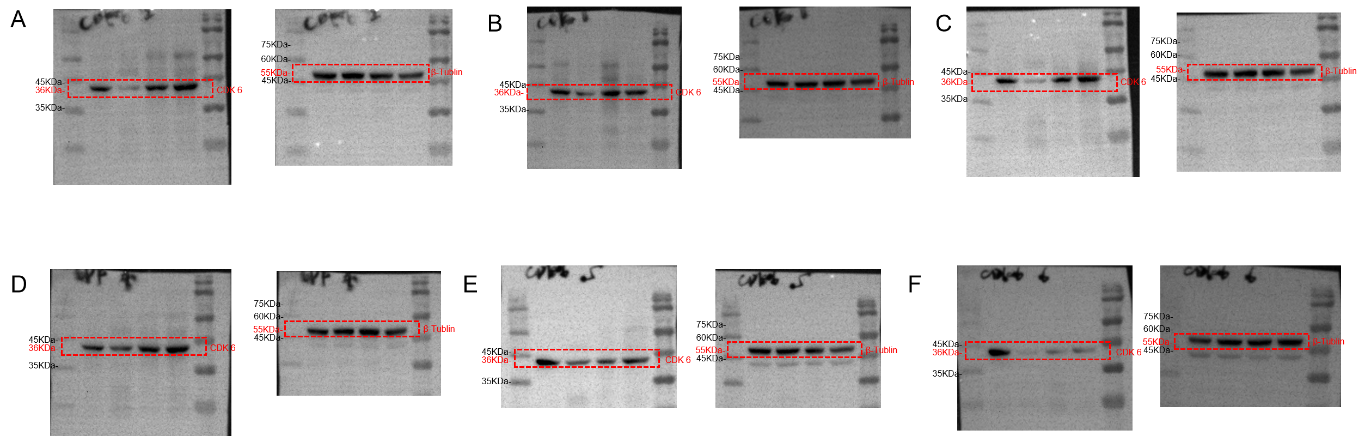


The raw data of CDK 6 & β-Tublin in Figure 7D. A. The original image of CDK 6 and β-Tublin in Figure 7D; B-F: Raw data of CDK6 and β-Tublin in Figure 7D.


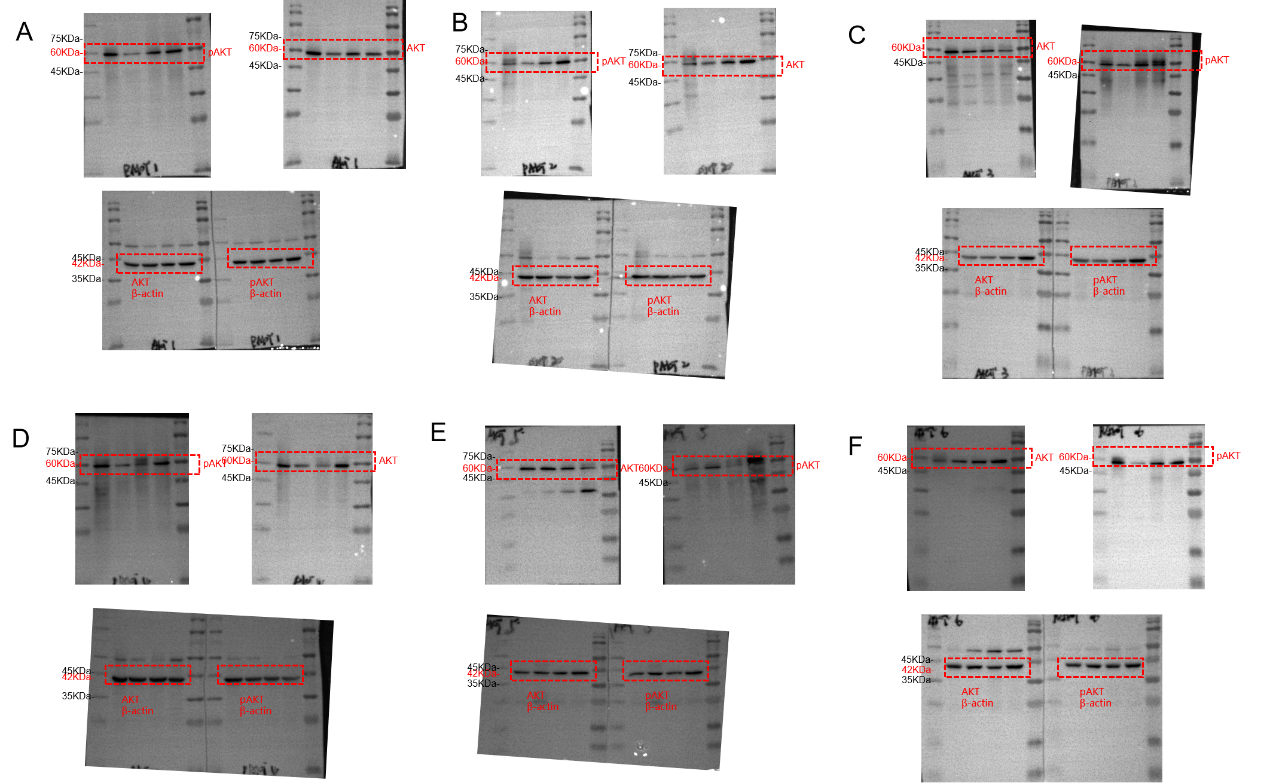


The raw data of p-AKT/AKT & β-Actin in Figure 8D. A. The original image of p-AKT/AKT & β-Actin in Figure 8D; B-F: Raw data of p-AKT/AKT & β-Actin in Figure 8D.


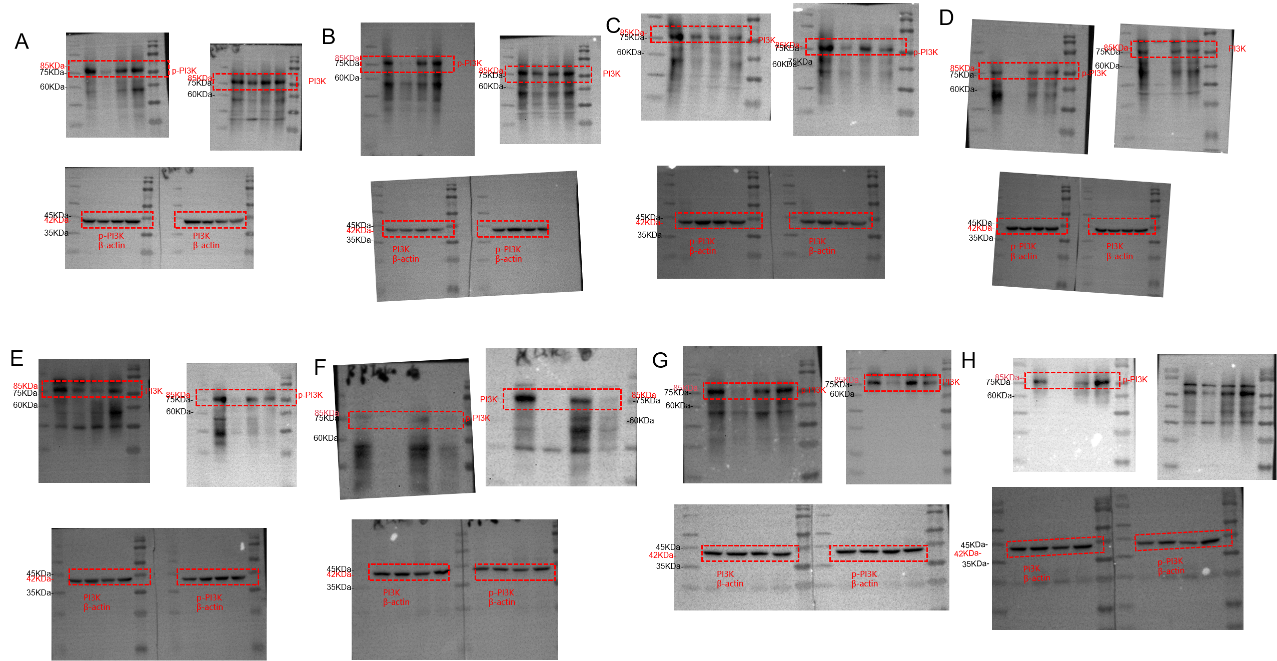


The raw data of p-PI3K/PI3K & β-Actin in Figure 8D. A. The original image of p- PI3K/PI3K & β-Actin in Figure 8D; B-F: Raw data of p- PI3K/PI3K & β-Actin in Figure 8D.
